# Enhanced circularly polarized luminescence attained *via* self-assembly of heterochiral as opposed to homochiral dipeptides in water†

**DOI:** 10.1039/d4sc01631a

**Published:** 2024-08-12

**Authors:** Sayan Bera, Umesh, Santanu Bhattacharya

**Affiliations:** a School of Applied and Interdisciplinary Sciences, Indian Association for the Cultivation of Science Kolkata 700032 India sb23in@yahoo.com sb@iisc.ac.in santanu.bhattacharya@iacs.res.in; b Department of Organic Chemistry, Indian Institute of Science Bangalore 560012 India; c Technical Research Centre, Indian Association for the Cultivation of Science Kolkata 700032 India; d Department of Chemistry, Indian Institute of Science Education and Research Tirupati 517619 India

## Abstract

Circularly polarized luminescence (CPL) is gaining interest across various disciplines, including materials science, pharmaceuticals, and sensing technologies. Organic molecules, due to their ease of synthesis and reduced toxicity, are a focus for achieving high dissymmetry values (*g*_lum_) in CPL. Here, we present a low molecular weight molecule (1), a dipeptide (Ala–Phe) covalently linked with tetraphenyl-ethylene (TPE), an Aggregation-Induced Emission luminophore (AIE-gen). Varying the stereochemistry of amino acid chiral centers, we synthesized homochiral 1-(l, l) & 1-(d, d) and heterochiral 1-(l, d) and 1-(d, l). In aqueous media, these molecules exhibit aggregation-induced chirality at the TPE chromophore. Heterochiral systems form sheet-like structures, displaying a bisignate induced circular dichroism signal and a good *g*_lum_ value for CPL [7.5 (±0.04) × 10^−3^]. Conversely, homochiral systems adopt fibrillar morphology, exhibiting a monosignate induced circular dichroism signal with a lower dissymmetry value for CPL [1.3 (±0.05) × 10^−3^]. This study introduces the concept of chiroptical amplification, emphasizing enhanced CPL through heterochiral peptide-induced CPL compared to its homochiral counterpart, with an ON and OFF CPL signal at low and high temperature respectively.

## Abstract 2

The dipeptide-based molecule 1 showed Circularly Polarized Luminescence (CPL) in aqueous media, with *g*_lum_ values increasing from 1.3 (±0.05) × 10^−3^ for the homochiral system to 7.5 (±0.04) × 10^−3^ for the heterochiral system.

## Graphical abstract

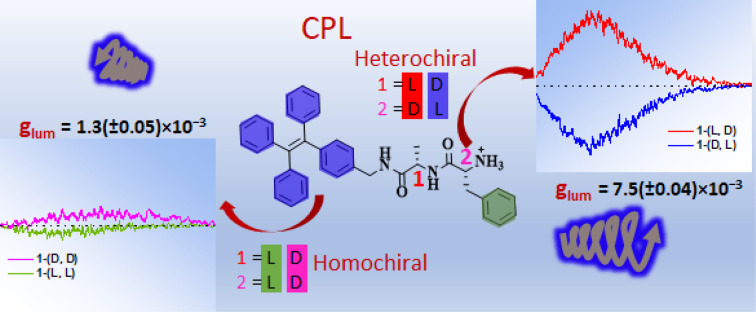

## Introduction

Chirality, which essentially stems from non-superimposable, mirror-image molecules, is a fundamental property of asymmetry that has profound and widespread applications in chemistry,^1^ biology,^2^ materials^3^ science *etc.* Chirality gives rise to chiroptical properties such as optical rotation, circular dichroism,^4^ and circularly polarized luminescence (CPL),^5,6^ in the fascinating realm of optical phenomena that have attracted increasing attention in recent years with various new applications realized across diverse scientific domains. Circularly polarized luminescence (CPL) is an intriguing excited state chiroptical property associated with chiral molecules and materials. The luminescence dissymmetry factor (*g*_lum_) is employed to quantify the CPL level. It is defined as 2(*I*_L_ − *I*_R_)/(*I*_L_ + *I*_R_), where *I*_L_ and *I*_R_ are the magnitudes of left- and right-circularly polarized emissions, respectively. The circularly polarized luminescence (CPL) phenomenon has attracted of late intense scientific interest driven by its versatile applications across a spectrum of disciplines that include materials science, pharmaceuticals, and sensing and imaging technologies.^7–9^ In this regard, numerous reports on the design and synthesis of luminescent chiral systems, comprising both organic^5^ and inorganic^10,11^ moieties have appeared in the literature. Organic molecules, in particular, have garnered significant research attention due to their ease of synthesis and low toxicity.^12–14^

Self-assembled chiral luminescent organic nanomaterials exhibit considerable promise in achieving tunability, responsiveness to stimuli, and enhancement of CPL properties.^15,16^ This potential arises from the existence of weak non-covalent interactions among the supramolecular nanostructures that may respond to various stimuli, including temperature, redox changes, pH variations, exposure to light *etc.*^17^ The value of *g*_lum_ for chiral small organic molecules falls typically within the range of 10^−5^–10^−4^ while the maximum possible |*g*_lum_| value is 2, which indicates completely left or right circularly polarized light. Therefore, recent research strategies such as assembly/aggregation, plasmon resonance, Förster resonance energy transfer, triplet–triplet annihilation photon up-conversion, liquid crystals, and intelligent molecular design are focused towards the amplification of the *g*_lum_ value.^18–21^ The molecular design of building blocks holds paramount importance in achieving high values of circularly polarized luminescence (CPL). Hence, several critical considerations emerge in the design process, encompassing the control of non-covalent interactions that govern self-assembly pathways, the strategic placement and number of chiral centers, as well as the selection and positioning of luminescent chromophores.^22^ Peptides being chiral and naturally occurring, emerge as notable candidates for the investigation of self-assembly in this context. They possess inherent chirality, and offer remarkable flexibility for the introduction of additional chiral centers by tuning the peptide sequence,^23–25^ facilitate straightforward covalent attachment to achiral luminophores,^26^ and often form nanoaggregates through self-assembly primarily driven by non-covalent interactions.^27–32^ Another crucial aspect is the enhancement of circularly polarized luminescence (CPL) in aqueous solutions, a development that may facilitate the utilization of CPL systems in selected biomedical applications.^33^ Further peptides, owing to their aqueous and biocompatibility, indeed present a promising avenue for exploration and achievement of this enhancement. The thermodynamic and kinetic factors that underpin the aggregation of peptides may determine whether they promote disease formation or they lead to therapeutic effects. Studies that shed light on self-assembly of short peptide segments or protein fragments in ageing and in related ailments are therefore important. In this regard, it is noteworthy to mention the pioneering work of Gazit *et al.* in comprehending the self-assembly mechanism of small peptides.^34^ In recent years, these authors demonstrated the involvement and utility of assemblies formed by small peptides in diverse applications.^35,36^

Herein, we report isomers of a molecule 1 (Table 1), which features a dipeptide system comprising stereogenically pure Ala–Phe dipeptides which is covalently attached to AIEgen luminophore known as tetraphenylethylene (TPE) to the C-terminal of this dipeptide. This strategic design not only enhances the water partitioning of the luminophore but also facilitates the self-assembly. What's particularly intriguing about our approach here is the covalent linking of dipeptides having amino acids with homo and hetero chirality with the AIEgen molecule. Specifically, we have introduced variations involving the two chiral centers present in the peptide, resulting in the creation of four distinct configurations: 1-(l, l), 1-(l, d), 1-(d, d), and 1-(d, l). Notably, the TPE AIEgen displayed a remarkable increase in the emission intensity within the heterochiral configurations *i.e.*1-(l, d) and 1-(d, l) surpassing that of the heterochiral configurations 1-(l, l) and 1-(d, d). This substantial enhancement ultimately resulted in a significant increase in circularly polarized luminescence (CPL) and the associated *g*_lum_ values, increasing from 1.3 (±0.05) × 10^−3^ for homochiral to 7.5 (±0.04) × 10^−3^ for the heterochiral system. The modulation of chiral centers could be a viable promising strategy for amplifying the *g*_lum_ values of chiral molecules, and we anticipate that our work will provide new research opportunities in this area.

**Table 1:** Molecular structure of the compounds used in the study. The variation of the -l/d stereochemistry of the amino acids used is described in the table for the molecule 1

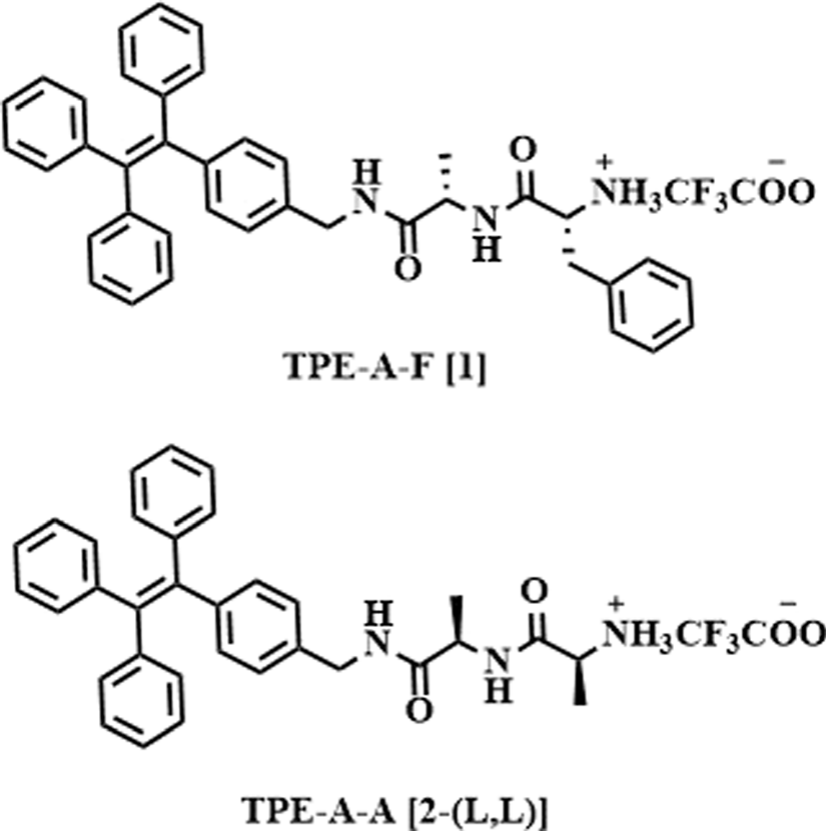		
Molecule (TPE-A-F)	Chirality@Ala	Chirality@Phe
1-(l, l)	l	l
1-(d, d)	d	d
1-(l, d)	l	d
1-(d, l)	d	l

## Results and discussion

The syntheses of four isomers of TPE linked 1 followed the procedures outlined in the Materials and methods section. In a concise summary, the individual chirally pure amino acids, alanine (l/d) and phenylalanine (l/d) were coupled using an EDC/DDC coupling reaction. Subsequently, a methyl ester deprotection step was performed to obtain a carboxyl (COOH) end group. This COOH group was then coupled in a similar manner using EDC/DCC coupling with the aminomethyl (NH_2_) group of the luminophore, TPE. The molecular structures of the molecules 1-(l, l), 1-(l, d), 1-(d, l), 1-(d, d), and 2-(l, l) are shown in Table 1. Initially, we examined the self-assembly of isomers 1 in aqueous media to examine the phenomenon of aggregation-induced emission of TPE when it is in an aggregated state. Subsequently, we conducted an in-depth spectroscopic analysis of the self-assembly for each isomer of the compound 1. Additionally, we employed a cryo-electron microscope (cryo-EM) to explore the morphological characteristics of the nanostructures formed by each isomer. The chiroptical properties were then investigated using circular dichroism (CD) and CPL spectrophotometers.

### Investigation of AIE properties of the stereoisomers of 1

**Fig. 1:**
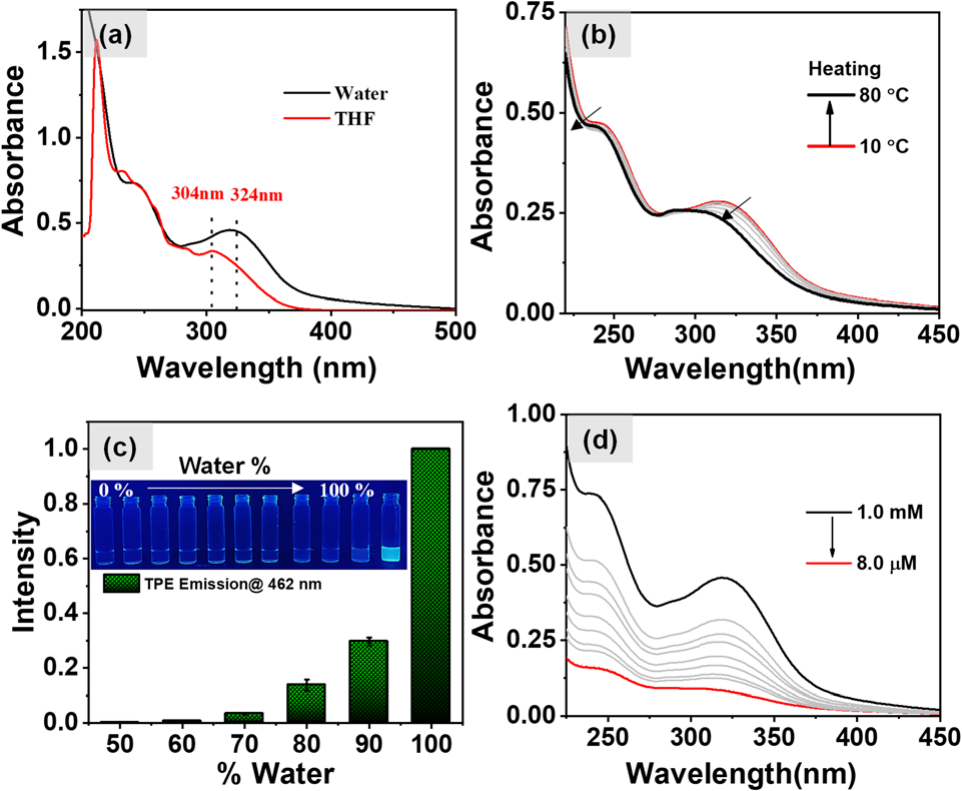
(a) UV-vis absorption spectra of 1-(l, d) in aqueous and THF medium (1 mM); (b) temperature dependent UV-vis spectra of 1-(l, d) in aqueous media exhibit the disassembly of the molecule at high temperature (0.5 mM); (c) normalized fluorescence intensity of 1-(l, d) with increasing water content in THF; (d) concentration dependent UV-vis spectra of 1-(l, d) in aqueous media exhibit only decrease in intensity upon dilutions.

Initially, UV-vis spectroscopy was carried out in a 1 mM THF solution of 1-(l, d) at a temperature of 25 °C. The results, as shown in Fig. 1a, exhibited the absorption bands at 304 nm and 285 nm, corresponding to the n–π* and π–π* transitions of the TPE molecule respectively.^37^ Additionally, an absorption band at 233 nm was observed, attributable to the π–π* transitions of the aromatic ring derived from the phenylalanine. Subsequently, UV-vis spectra for aqueous solutions of 1-(l, d) were taken which exhibited a noticeable red shift in the previously mentioned absorption bands to 324 nm, 298 nm, and 242 nm. This observation of red shift strongly suggests the formation of aggregated states in the aqueous media, with TPE forming J-aggregates as evidenced from the red shifted absorption band from 304 nm to 324 nm. The temperature-dependent UV-vis spectra offer valuable insights into the thermal stability of aggregates formed by self-assembly of 1-(l, d). When the aqueous solution of 1-(l, d) (*c* = 0.5 mM) was heated to 80 °C, the absorption bands at 324 nm and 242 nm displayed noticeable blue shifts, as shown in Fig. 1b. Remarkably, upon subsequent cooling of the sample to 10 °C, these absorption bands returned to their original positions (Fig. S1a†). This observation indicates the disassembly of aggregates at higher temperatures and the subsequent reformation of the same aggregates upon cooling. We recorded the fluorescence spectra for the aqueous solutions of 1-(l, d) (0.5 mM) under excitation at 340 nm and at a temperature of 25 °C. The results, shown in Fig. S1b,† revealed an emission peak centered at 462 nm. Notably, no emission was detected in the THF solution of 1-(l, d) due to the presence of monomeric TPE. However, as the percentage of water in the THF solution increased, the emission intensity exhibited a corresponding rise, as depicted in Fig. 1c. These observations validate the occurrence of the aggregation-induced emission phenomenon originating from the TPE luminophore which was further corroborated by the temperature dependent fluorescence spectral studies. The results of which are shown in Fig. S2a† which exhibit a gradual decrease in the emission intensity of TPE with the increase in temperature due to disassembly of the aggregates which further reappears on cooling (Fig. S2b†) of the sample due to the aggregated state of 1-(l, d). After the confirmation of AIE properties of 1 and its disassembly of the aggregates at higher temperature our next aim was to investigate the effect of dilutions on the self-assembled aggregates. However, as evidenced from the UV-vis spectra (Fig. 1d) and the fluorescence spectra (Fig. S1c and d†), there was no discernible shift in the absorption and emission spectral bands upon dilution. Instead, the observed change was a decrease in the intensity, attributable to the reduction in the concentration of the aggregated state suggesting no effect of dilutions on the aggregation induced emission properties of self-assembled aggregates.

### Investigation of the self-assembly behavior of 1

**Fig. 2:**
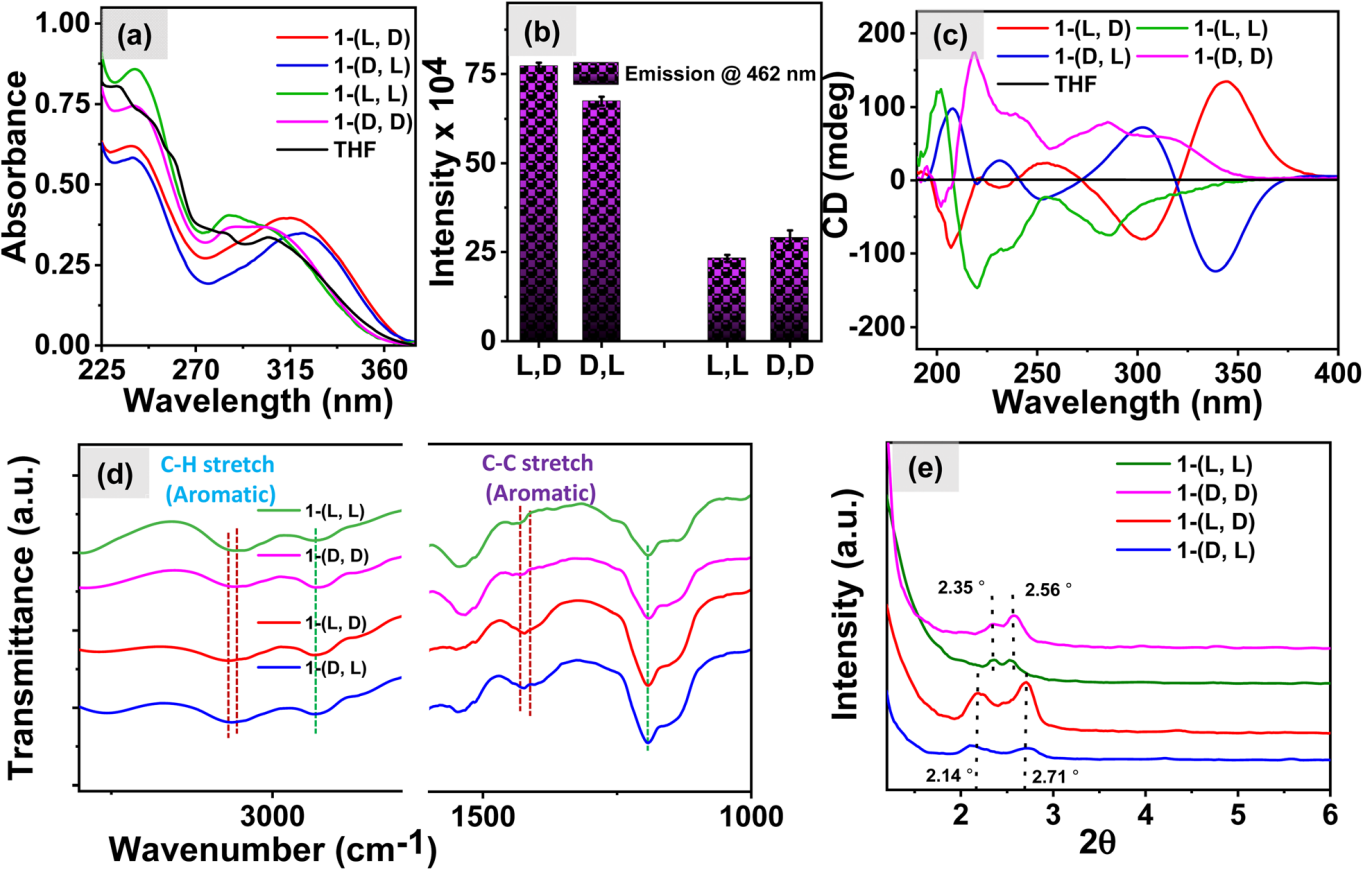
(a) UV-vis spectra of different isomers in aqueous media and THF (*c* = 0.5 mM); (b) bar diagram showing the fluorescence intensity of different isomers in aqueous media; (c) circular dichroism spectra exhibit the induced chirality in the TPE region of the heterochiral and homochiral systems, (d)FTIR spectra, where red lines demonstrate the shift in peaks and green dotted lines demonstrate no shift, and (e) XRD patterns for the assemblies of heterochiral and homochiral molecules respectively.

The role of chiral centers in the self-assembly behavior of the compound 1 was investigated using various spectroscopic techniques. Initially, the UV-vis spectra shown in Fig. 2a reveal that in the aqueous environment, the compounds with homochirality, 1-(l, l) and 1-(d, d), exhibit absorption bands at 307 and 285 nm, which correspond to the absorbance of the TPE moiety and are similar to the absorption of monomeric TPE in a THF solution. However, the peak corresponding to the aromatic segment of the peptide at 242 nm was red-shifted for both 1-(l, l) and 1-(d, d) molecules in the aqueous solution with respect to the THF solution peak at 233 nm, indicating the presence of dipeptide molecules in the self-assembled state. Interestingly, the molecules 1-(l, d) and 1-(d, l) in aqueous media exhibited red-shifted absorption spectral bands, suggesting the presence of an aggregated state of TPE in these systems.

To corroborate these findings, we examined fluorescence spectra, and the results presented in Fig. 2b and S2c† demonstrate significantly higher emission intensity of TPE in the heterochiral system compared to that in the homochiral system. This observation implies the presence of a higher number of TPE aggregates in the heterochiral system and hence this is also consistent with the conclusions drawn from the UV-vis studies. Furthermore, to delve deeper into the mechanism of self-assembly of 1 we recorded CD spectra. The results shown in Fig. 2c exhibit CD signals with the mirror image for the respective isomers of 1-(l, l) and 1-(d, d) and the isomers of 1-(l, d) and 1-(d, l). However, on examining the CD spectra we observed a bisignate signal characterized by CD peaks at 340 nm and 304 nm for the heterochiral molecules in the TPE region. In contrast, homochiral molecules exhibit a CD signal at 288 nm with a shoulder at 325 nm attributed to the TPE luminophore. These findings suggest the presence of well-organized J-aggregates of the TPE molecule in the heterochiral systems which aligns well with the observations from the UV-vis studies, leading to exciton coupling through space, which is responsible for the manifestation of the bisignate CD signal. In contrast, the absence of organized TPE aggregates in homochiral systems results in the lack of exciton coupling and, consequently, degenerate excited states.^38–40^ Furthermore, the FTIR spectra shown in Fig. 2d exhibit a shift in the peaks corresponding to the aromatic C–H stretch from 3056 cm^−1^ in the heterochiral system to 3066 cm^−1^ in the homochiral system. Additionally, the aromatic C–C stretch shifts from 1430 cm^−1^ in the heterochiral system to 1409 cm^−1^ in the homochiral system.^41^ The manifestation of such shifts suggests the presence of TPE stacks in the heterochiral system. The small-angle X-ray diffraction (SAXRD) patterns shown in Fig. 2e exhibited well-defined diffraction peaks for the assemblies at 2*θ* of 2.71 for the heterochiral system and 2.56 for the homochiral system, with corresponding *d*-spacing values of 3.26 and 3.45 nm, respectively. The molecular length of 1 based on the CPK model was estimated to be 1.73 nm (Fig. S3a†). The *d*-spacing values are more than the molecular thickness and less than twice the molecular thickness for the heterochiral system, suggesting the formation of an interdigitated bilayer structure. In contrast, the *d*-spacing (the distance between successive, parallel planes of atoms) values for the homochiral system are similar to twice the molecular thickness, indicating a head-to-tail packing of the bilayer structure.^41–43^ The proposed hydrogen bonding and molecular packing are illustrated in Fig. S3b,† using the ChemDraw model of the molecule derived from the optimized geometry shown in Fig. S4.† In the heterochiral system, phenylalanine forms a stack with TPE molecules. However, in the homochiral system, no such interaction is observed between the phenylalanine and TPE molecules. These observations suggest that π-stacking between the phenyl and TPE results in the bisignate CD signal as well as enhanced emission for the heterochiral molecules. Furthermore, to understand the role of the chiral centers in determining the chiroptical properties of nanostructures, we investigated the self-assembly behavior of the molecule 2-(l, l) in aqueous media. The UV-vis spectra shown in Fig. S5a† indicate that the absorption corresponding to the TPE moiety remains similar to that of the monomeric TPE as was observed in 1-(l,l) in THF. However, for the aggregated TPE, the absorption maximum was red-shifted in 1-(l,l) in an aqueous media. The emission spectra in Fig. S5b† revealed a lower emission intensity for 2-(l, l) compared to the emission of the TPE in an aggregated state for 1-(l, l). Interestingly, the CD spectra in Fig. S5c† displayed a weak negative CD signal at 227 nm for the 2-(l, l) assembly in an aqueous media, suggesting the presence of β-sheet like nanostructures. These observations imply that the phenylalanine centre plays a crucial role in regulating the chiroptical properties of the nanostructures.

**Fig. 3:**
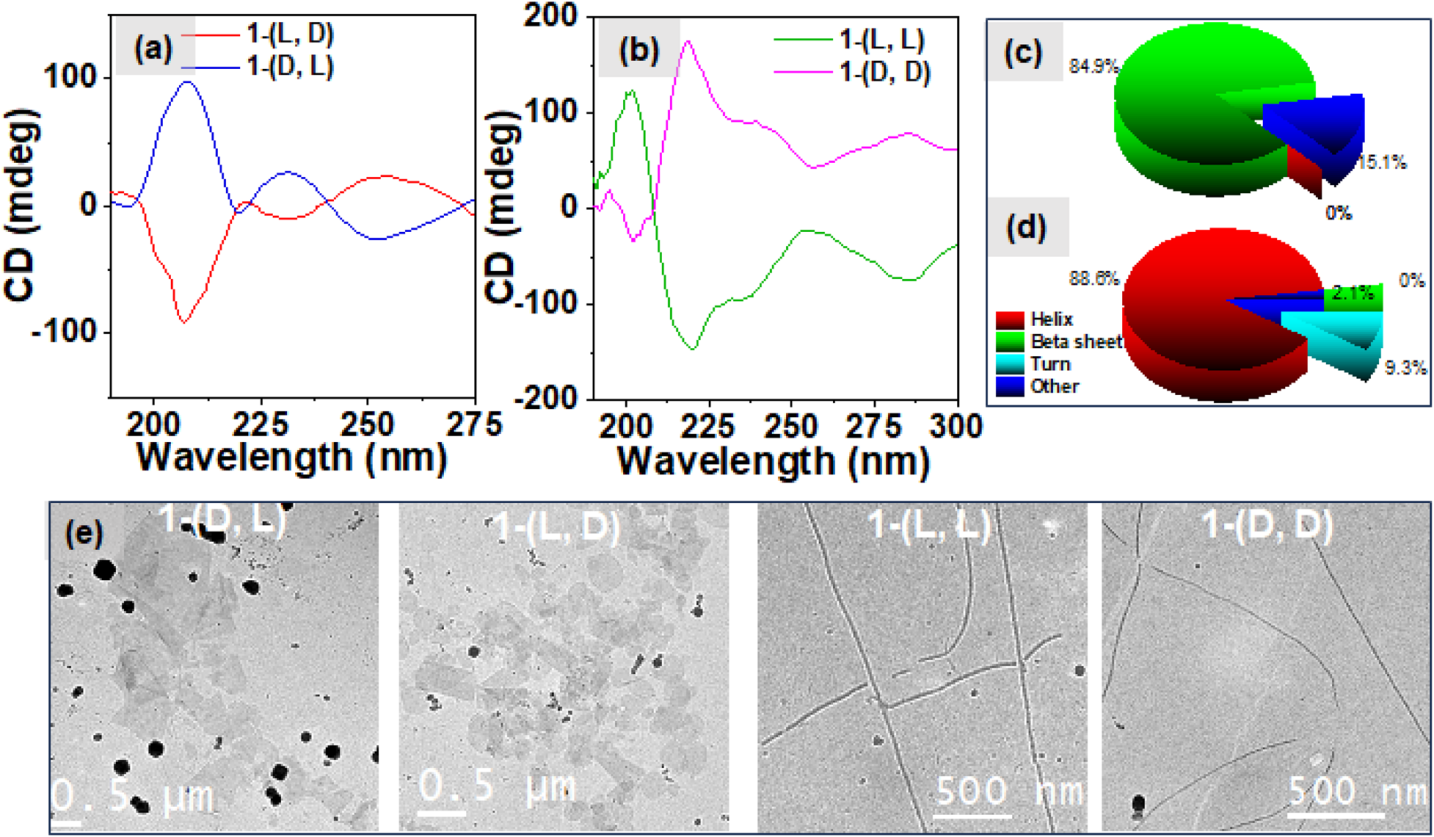
The circular dichroism spectra showing the formation of secondary structures due to self-assembly of (a) heterochiral and (b) homochiral systems; pie chart depicting the percentage of secondary structures present in the (c) heterochiral and (d) homochiral systems; (e) Cryo-EM images of the different systems showcasing the sheet like morphology for heterochiral systems and fibrillar morphology for the homochiral systems.

The amide region of the CD spectra, from 190 to 250 nm, offers valuable insights into the formation of secondary structures during peptide self-assembly. Fig. 3a exhibits negative CD signals at 209 and 230 nm for 1-(l, d) and as expected its enantiomer 1-(d, l) exhibits positive CD signals at 209 and 230 nm respectively which suggest the formation of β-sheet secondary structures in the heterochiral system. Conversely, in the case of the homochiral system (as depicted in Fig. 3b), we observed a negative signal at 218 nm with a shoulder at 230 nm and a negative CD signal at 200 nm for 1-(l, l) and the mirror image for its enantiomer 1-(d, d), which correspond to the formation of α-helix secondary structures in the self-assembly of the homochiral system.^37,44–46^ The deconvolution analysis of these CD spectra, as depicted in Fig. 3c for the heterochiral system and Fig. 3d for the homochiral system, revealed the composition of secondary structures. In the heterochiral systems, approximately 84.9% of the secondary structures in the self-assembly were identified as β-sheets, with the remaining 15.1% categorized as other or random structures. Conversely, for the homochiral system, the analysis indicated that approximately 88.6% of the secondary structures were helices, 9.3% were turns, and 2.1% were other structures. The concentration dependent CD spectra shown in Fig. S8a and b† for the heterochiral and Fig. S8c and d† for the homochiral system exhibit no change in the spectral band shapes whereas the ellipticity decreases while reducing the concentration indicating the associative nature of the self-assembly of 1. To further explore the morphology of the nanostructures formed by various isomers of compound 1, we employed Cryo-EM microscopy. The micrographic images displayed in Fig. 3e illustrate the formation of fibrils in the case of the homochiral system. In contrast, the heterochiral systems exhibited formation of randomly distributed 2D-sheets which was further supported by the SEM images shown in the Fig. S6.† The nanofibers formed by 1-(l, l) displayed right-handed (P-type) twists, while those formed by 1-(d, d) exhibited left-handed (M-type) twists, as shown in the Cryo-EM images given in Fig. S7†.^47^ This divergence in nanostructure morphology highlights the distinct self-assembly mechanisms of systems with homochiral centres compared to those with the heterochiral centres, as observed through spectroscopic techniques. The “like–like” intermolecular interaction involved in case of the assemblies of homochiral systems might be responsible for the formation of the robust fibrillar morphology whereas “like–unlike” intermolecular interaction in the case of the assemblies of the heterochiral system may be responsible for the formation of the brittle sheet like structure.^46,48^

### Temperature dependence of self-assembly

**Fig. 4:**
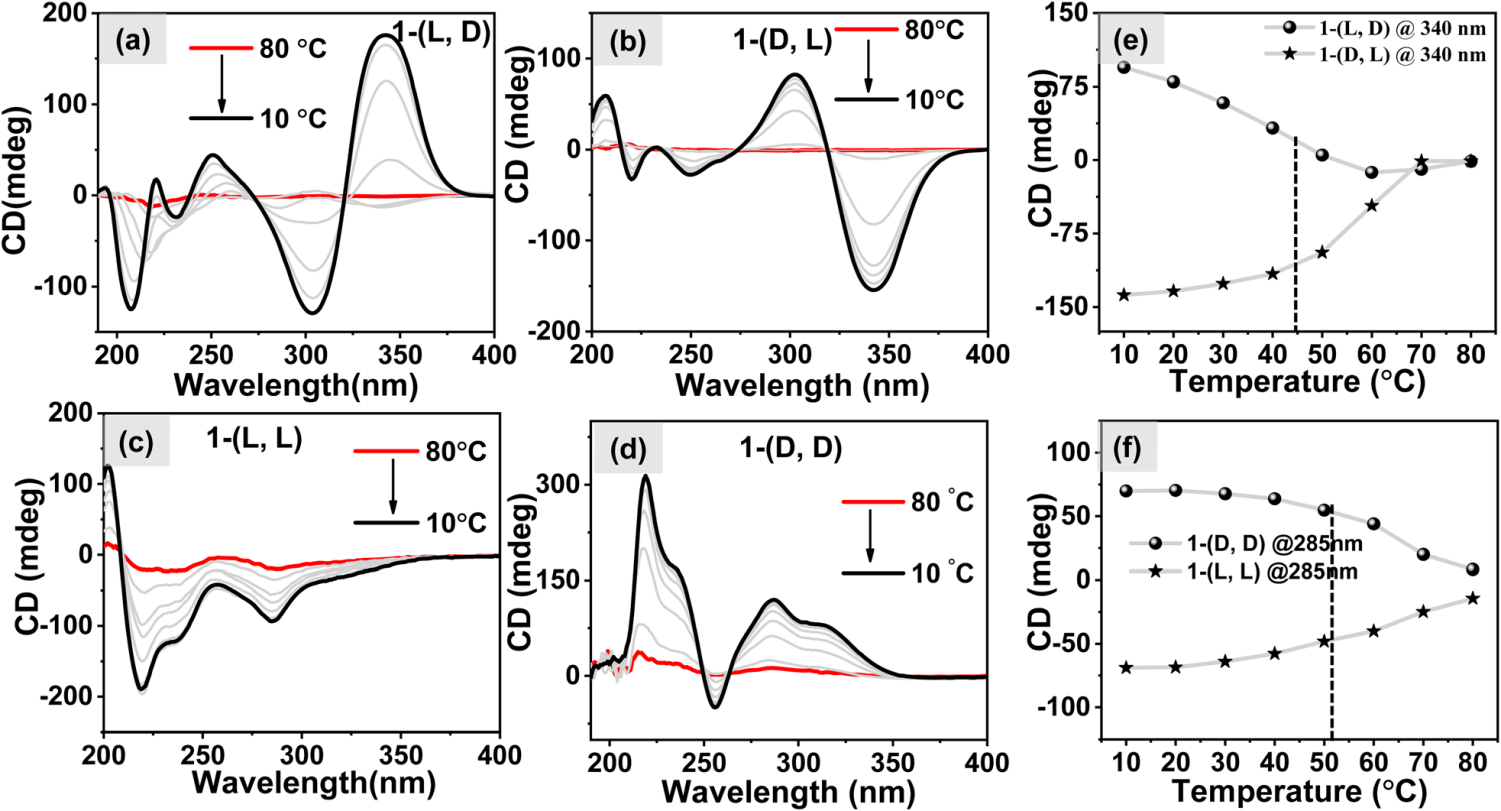
The variations observed in the CD spectra of heterochiral systems (a and b) and homochiral systems (c and d) during cooling of aqueous solution of molecules; (e) the m.deg value @340 nm *vs.* temperature plot for the heterochiral system, and (f) the m.deg value @285 nm *vs.* temperature dependent plot for the homochiral systems.

To investigate the response of self-assembled nanostructures towards the temperature variations, we recorded temperature-dependent CD spectra. This involved heating and cooling an aqueous solution containing 1 (*c* = 1 mM). The results shown in Fig. S9a–S9d† show that both systems follow a gradual decrease in the intensity of CD signals as the temperatures increase. At higher temperatures, they practically turn CD silent, indicating the transition to monomeric forms of molecules. Upon cooling the samples, the CD signal intensity gradually increased, signifying the reformation of self-aggregated states by all the molecules as shown in Fig. 4a–d. Notably, when analyzing the m.deg @340 nm *vs.* temperature plot for the heterochiral system (Fig. 4e) and the m.deg @285 nm *vs.* temperature plot for the homochiral system (Fig. 4f), we observed that the melting temperatures (*T*_m_) for the self-assembled structures were ∼45 °C and ∼51 °C, respectively. This suggests that the fibril nanostructures remain stable at higher temperatures compared to the 2D sheets. The disparity in the melting point of these two distinct systems may be explained by considering the “like–like” and “like–unlike” intermolecular interaction which played a vital role in the formation of different nanostructures.^47–49^

### Investigation of CPL properties

**Fig. 5:**
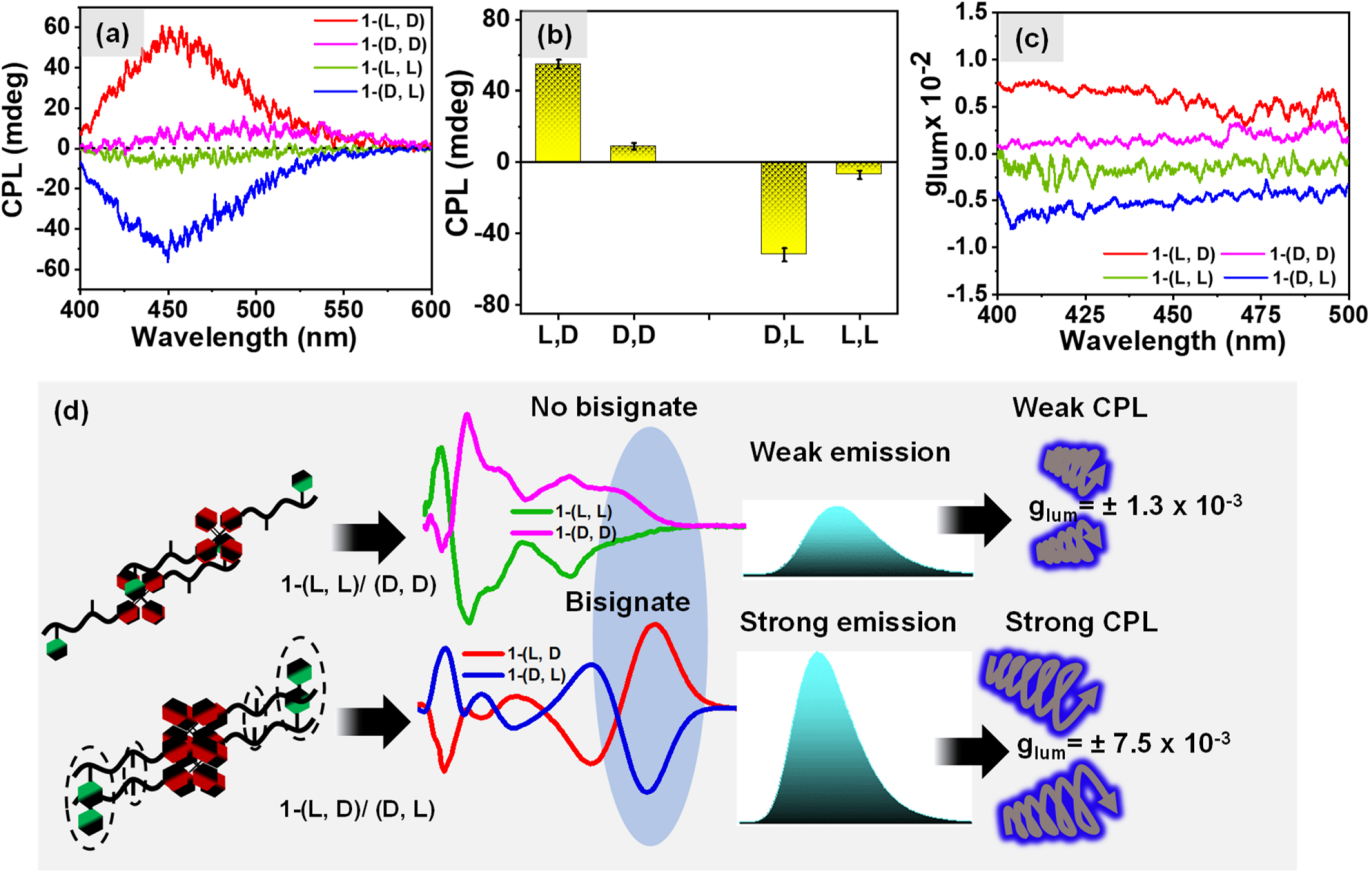
(a) CPL spectra of homochiral & heterochiral aggregates in aqueous solution (*λ*_ex._ = 340 nm) under ambient conditions; (b) bar-diagram showing the observed *g*_lum_ values @450 nm for the homochiral and the heterochiral aggregates; (c) CPL dissymmetry factor (*g*_lum_) *vs.* wavelength plot for the different systems, and (d) schematic illustration of the observed amplified CPL and *g*_lum_ for heterochiral systems as compared to the homochiral systems.

**Fig. 6:**
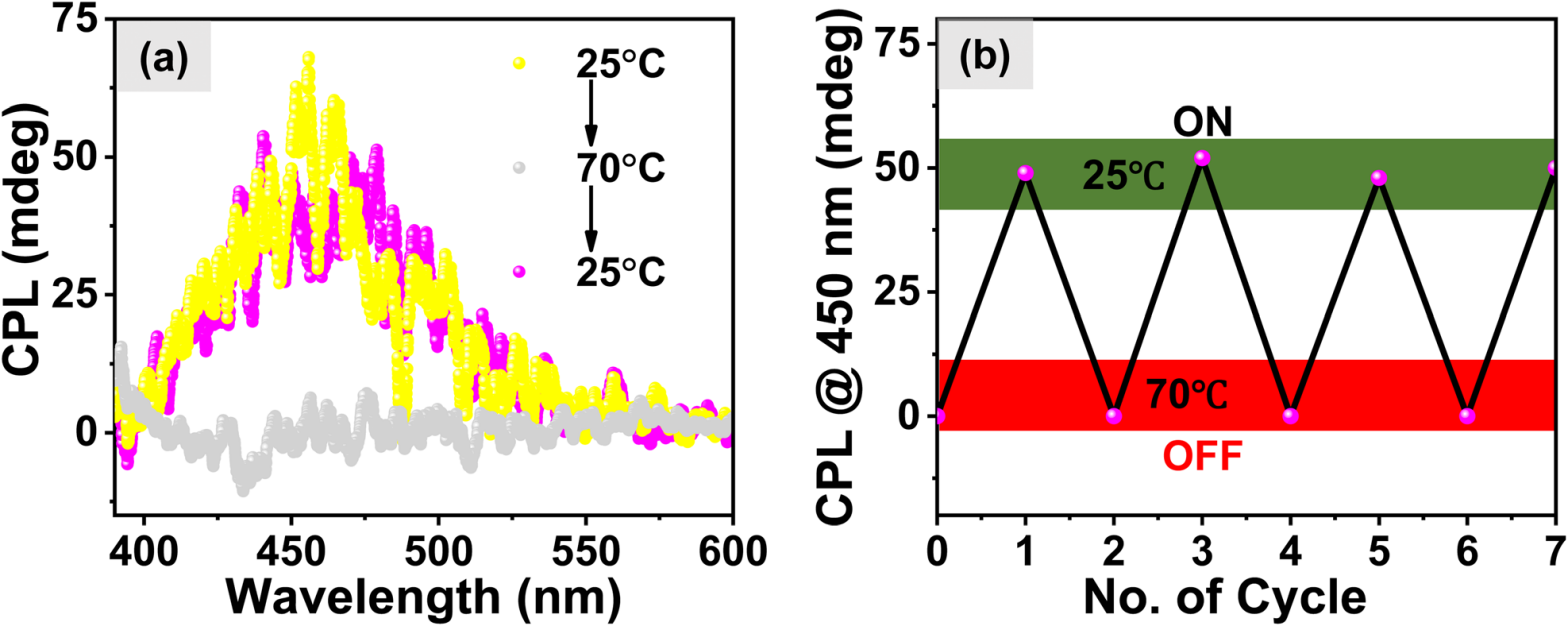
(a) Temperature dependence of CPL of 1-(l, d) and (b) m.deg values of 1-(l, d) after heating–cooling cycles; CPL On and OFF was observed at 25 and 70 °C respectively, due to disassembly at higher temperature. CPL was stable even after 4 heating–cooling cycles.

The influence of changing the stereochemistry of a chiral center on the self-assembly of the dipeptide molecule was investigated which led to the formation of organized aggregates of the AIEgen TPE luminophore followed by amplification of the emission of TPE. Our next objective was to investigate the influence of hetero and homo-chirality in dipeptide on the amplification of its excited state chiroptical properties. In this regard, we measured the circularly polarized luminescence (CPL) of aqueous solution of different isomers of 1 (0.5 mM). The results shown in Fig. 5a exhibit a negative and positive circularly polarized luminescence (CPL) for the molecules 1-(l, l) and 1-(d, d) respectively. Interestingly, an 8-fold amplification of the CPL intensity was observed for the heterochiral molecules, 1-(d, l) and 1-(l, d) as shown in Fig. 5b which is however attributed to the bisignate signal originating from the exciton coupling through space due to organized J-aggregates of the TPE luminophore. The corresponding DC values for the CPL spectra are shown in Fig. S10a.† The degree of circularly polarized luminescence (CPL) of the compound was evaluated from a constant called luminescence dissymmetry factor (*g*_lum_). Generally, *g*_lum_ is defined using the equation below*g*_lum_ = [*θ* × 6.9813 × 10]^−5^/*I*where *I* denotes the total luminescence intensity during the CPL measurement and *θ* is the ellipticity.^50^ The *g*_lum_ values observed for different isomers of 1 in aqueous media are shown in Fig. 5c which also exhibits the significant amplification of *g*_lum_ values for heterochiral molecules with the observed *g*_lum_ value of 7.5 (±0.04) × 10^−3^ in comparison of the homochiral molecules with the *g*_lum_ value of 1.3 (±0.05) × 10^−3^. Comparison of different circularly polarized luminescent materials with reported *g*_lum_ values is shown in Table S1 (ESI).† In prior experiments, we explored the temperature-dependent self-assembly of 1-(l, d). Additionally, the investigation extended to the Circularly Polarized Luminescence (CPL) emanating from the molecules as the temperature was increased. The results shown in Fig. 6a revealed the absence of CPL at 70 °C. Intriguingly, upon subsequent cooling to 25 °C, the CPL signal was recovered, imparting a distinctive ON and OFF CPL phenomenon at low and high temperatures, respectively. We have observed a similar ON-OFF behavior for the homochiral molecules upon heating and cooling the sample. However, the change was not as prominent as in the heterochiral molecule assemblies (Fig. S10b†). Fig. 6b highlights the reproducibility of the Circularly Polarized Luminescence (CPL) signal, which persisted even after subjecting the system to four consecutive heating–cooling cycles.

## Experimental

### General information

All chemicals, solvents, and silica gel for TLC were obtained from well-known commercial sources and were used without further purification. Distilled and freshly dried solvents were used for the column chromatography.

### NMR, FT-IR, and ESI-MS experiments

^1^H-NMR spectra were recorded on a FT-NMR Bruker DPX 400 MHz NMR spectrometer. The ^13^C-NMR spectra were recorded at 100 MHz using the same NMR spectrometer. Chemical shifts were reported in ppm relative to that of TMS as an internal standard. FT-IR spectra were recorded on a PerkinElmer 100 FT-IR spectrometer. An electrospray ionization (ESI) Q-TOF mass spectrometer was used for the mass spectrometric analysis of the compounds.

### Preparation of the experimental samples

0.58 mg of each isomer of 1/TPE-A-F was weighed accurately in a glass vial and then 1 mL of Milli-Q water was added and the sample was heated at 90 °C for 5 min and then bath sonicated for 5 min followed by cooling to room temperature. This cycle was repeated 5 times for the sample preparations (concentration = 1 mM).

### UV-vis and fluorescence spectroscopy

The UV-spectra and the fluorescence spectra of various samples were collected using a Shimadzu UV-2600 UV-VIS spectrophotometer and Horiba Fluoromax-4 spectrofluorometer respectively. Quartz cuvettes with 1 mm and 10 mm path lengths were used for the experiments for UV and fluorescence measurements respectively. Temperature-dependent experiments were done in a similar setup where a Julabo 300F heating circulator was attached to the sample holder to control the temperature.

### Circular dichroism measurements

The circular dichroism measurements were done on a JASCO J-1500 spectrophotometer. The circular dichroism spectra of various samples were recorded using a 1 mm quartz cuvette with a scan rate of 100 nm min^−1^. Temperature-dependent circular dichroism spectral studies were performed using a Peltier controlled sample holder. CD multivariate analysis was performed to calculate the relative percent of the secondary structures using the software provided by the JACSO corporation (JWMVS-529 Multivariate SSE, JASCO referenced with 26 proteins).^51^ During the analysis the 190–280 nm model was chosen and both the Principal Component Regression (PCR) and Partial Least Squares (PLS) models were used for the analysis.

### Circularly polarized luminescence measurements

The circularly polarized luminescence spectra were recorded with a 1 mm quartz cuvette on a CPL-300 spectrophotometer supplied by the JASCO corporation employing multiple spectral accumulations.

### Cryo-electron microscopy (Cryo-EM)

For the Cryo-EM analysis 1 mL of the sample (concentration = 1 mM) was prepared as per the procedure described before. From this stock solution ∼3 μL of the sample was drop-coated on a carbon coated Cu TEM grid (300 mesh). The grid was supported on a Gatan Cryo Plunging system (GATAN CP3). The sample-cast grid was then cryo quenched into liquid ethane to form a layer of ethane. The specimen was then quickly transferred to a cryo-cooled sample holder. After transferring the specimen to the column of a Cryo-EM, the specimen was then examined with a cryo-cooled objective lens at 120 kV electron acceleration voltage.

### FE-SEM

For the SEM analysis of the aggregates we drop casted the sample in a clean glass substrate. The sample was dried at room temperature under ambient conditions followed by drying in a vacuum for 6 h. The prepared specimen was coated with Pt for 90 seconds and then observed using a JEOL JSM-7500F field emission scanning electron microscope.

### Small-angle X-ray diffraction (SAXRD)

For the small angle XRD analysis of the sample we drop-casted the samples on a pre-cleaned glass substrate to prepare a film. The obtained film was dried under ambient conditions first followed by drying in a vacuum. The XRD measurement was done on a Bruker D8 Advance A25 model (Operating voltage and current: 40 kV and 40 mA respectively) with a scan speed of 1 second per step.

### DFT minimization

The different compound structures were drawn using GaussView 6.0 Software. The structures of the isomers were optimized with the B3LYP/6-31G level of theory (water, PCM solvation model) using the gaussian 16 package.

## Conclusions

In this study, we examined the enhancement of circularly polarized luminescence (CPL) in aqueous media through molecular self-assembly and chirality variation. We synthesized four stereoisomers of the same molecule, 1, comprising dipeptides of Ala–Phe amino acids the C-termini of which were covalently attached with an AIEgen luminophore, tetraphenylethylene (TPE). This unique design not only improved the water solubility of the luminophore but also promoted the formation of different motifs of the self-assembly. Our approach introduced variations in the molecule involving hetero and homo chiral centers, resulting in four distinct configurations: 1-(l, l), 1-(l, d), 1-(d, d), and 1-(d, l). Our investigation delved into the self-assembly behavior of the isomeric molecules in aqueous solutions. Spectroscopic techniques, including UV-vis, fluorescence, and circular dichroism (CD) spectroscopy, provided pertinent insights into the formation of the aggregated states, secondary structures, and the distinct nanostructured morphologies adopted by the homo and hetero chiral systems. Remarkably, the heterochiral configurations 1-(l, d) and 1-(d, l) exhibited significantly enhanced emission intensity compared to their homochiral counterparts 1-(l, l) and 1-(d, d). Cryo-EM microscopy confirmed the formation of fibrils in the aggregates of the homochiral systems and 2D-sheets in the corresponding heterochiral systems, highlighting the unique self-assembly mechanisms at play. Temperature-dependent CD spectral studies demonstrated the thermal responsiveness of the self-assembled nanostructures, with the melting temperatures (*T*_m_) for the self-assembled structures being 45 °C and 51 °C for the heterochiral and homochiral systems, respectively. This indicates the superior thermal stability of the fibrillar nanostructures in comparison to that of the 2D sheets. Finally, our investigations into the circularly polarized luminescence (CPL) properties reveal an ∼8-fold amplification of the CPL intensity in the heterochiral molecules, attributed to the formation of organized J-aggregates of the TPE luminophore. A substantial increase in the circularly polarized luminescence (CPL) and the luminescence dissymmetry factor (*g*_lum_) values, elevating from 1.3 (±0.05) × 10^−3^ for the homochiral to 7.5 (±0.04) × 10^−3^ for the heterochiral system, along with a ON and OFF CPL signal at low and high temperature, respectively, underscores the potential of molecular design and chirality variation in amplifying the CPL properties of isomeric molecules.

Overall, the present work not only advances the understanding the role of chirality in peptide assemblies but also offers a promising avenue for the design and engineering of chiral nano-biomaterials with enhanced CPL properties. This research opens up hitherto unseen opportunities for the application of chiral nanomaterials in various scientific knowledge domains, including materials science, biopharmaceutics, and sensing technologies. Work is currently underway to achieve stimuli responsive CPL in aqueous media from sequence-specific peptide-based molecules in our laboratory.

## Author contributions

Sayan Bera and Umesh contributed equally.

## Conflicts of interest

There are no conflicts to declare.

## Supplementary Material

SC-015-D4SC01631A-s001

## Data Availability

All the data supporting this article have been included in the main text and ESI.†
