# Supplementary material for: Enhanced circularly polarized luminescence attained via self-assembly of heterochiral as opposed to homochiral dipeptides in water
Source: Chem Sci. 2024 Aug 12;15(34):13987–97. doi: 10.1039/d4sc01631a (PMC11320125; doi:10.1039/d4sc01631a)
Supplement: SC-015-D4SC01631A-s001 [file SC-015-D4SC01631A-s001.pdf]

Electronic Supplementary Material (ESI) for Chemical Science.
This journal is © The Royal Society of Chemistry 2024

Supporting Information

Enhanced Circularly Polarized Luminescence Attained via Self-assembly of Heterochiral as opposed to Homochiral Dipeptides in water

Sayan Bera, [a],† Umesh, [a], † Santanu Bhattacharya*, [a,b,c,d]

[a] *Mr. Sayan Bera, Mr. Umesh, Prof. Dr. Santanu Bhattacharya*
School of Applied and Interdisciplinary Sciences
Indian Association for the Cultivation of Science
Kolkata 700032, India.
E-mail: sb23in@yahoo.com, sb@iisc.ac.in, santanu.bhattacharya@iacs.res.in

[b] *Prof. Santanu Bhattacharya*
Department of Organic Chemistry
Indian Institute of Science Bangalore
560012, India.

[c] *Prof. Santanu Bhattacharya*
Technical Research Centre
Indian Association for the Cultivation of Science
Kolkata 700032, India.

[d] *Prof. Santanu Bhattacharya*
Department of Chemistry
Indian Institute of Science Education and Research Tirupati
517619, India.

† These authors contributed equally.

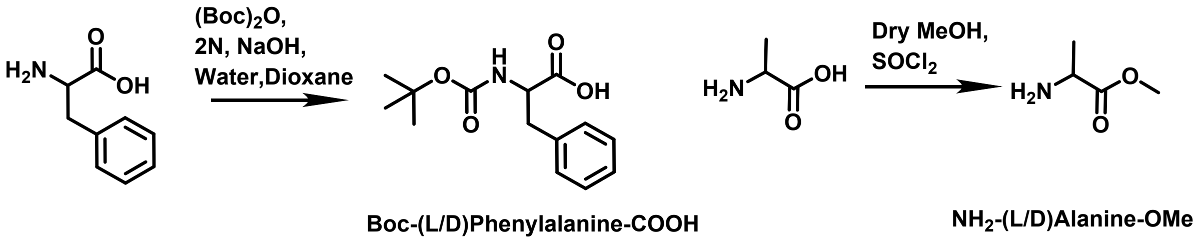

Scheme-1: Scheme for synthesis of Boc-Phenylalanine-COOH and NH_2-Alanine-OMe.

Synthetic Methods:

Synthesis of Boc-Phenylalanine-COOH:

L/D-Phenylalanine (5 g, 0.03 mmol) was taken in a 250 mL round bottom flask and solubilized by adding 20 mL of 4N NaOH solution in water. To this a dioxane solution of $(Boc)_2O$ (8.34 mL, 0.036 mmol) was added dropwise using dropping funnel. The resulting solution was then stirred overnight at room temperature. After this the reaction mixture was acidified with 1N HCl solution to a pH~5-6 and extracted with EtOAc (3 x 200 ml). This afforded a yellowish oil as product, which was characterized by ^{1}H NMR and used for the next step without further purification.[1, 2]

^{1}H NMR (400 MHz, $CDCl_3$, δ ppm): 1.31–1.42 (m, 9H), 3.19–3.23 (m, 2H), 6.65 (br, 1H), 4.94 (br, 1H), 7.19 (mc, 2H), 7.26–7.31 (m, 3H).

Synthesis of NH_2-Alanine-OMe:

The compound NH_2-Alanine-OMe was synthesized following a reported protocol. L/D-Alanine (2 g, 0.02 mmol) was dissolved in 10 mL of dry methanol. The solution was kept in an ice-bath and then 4 mL of $SOCl_2$ was added dropwise to the solution. The resulting solution was stirred at room temperature for 4 h. The solution was then dried under vacuo to get a yellowish transparent oil as product. The product was used without further purification.[3]

^{1}H NMR (400 MHz, $CDCl_3$, δ ppm): 1.27 (d, 3H), 3.56 (m,1H), 3.61 (s, 3H), 8.76 (br, 3H)

Scheme-2: Synthetic routes to various dipeptide analogues.

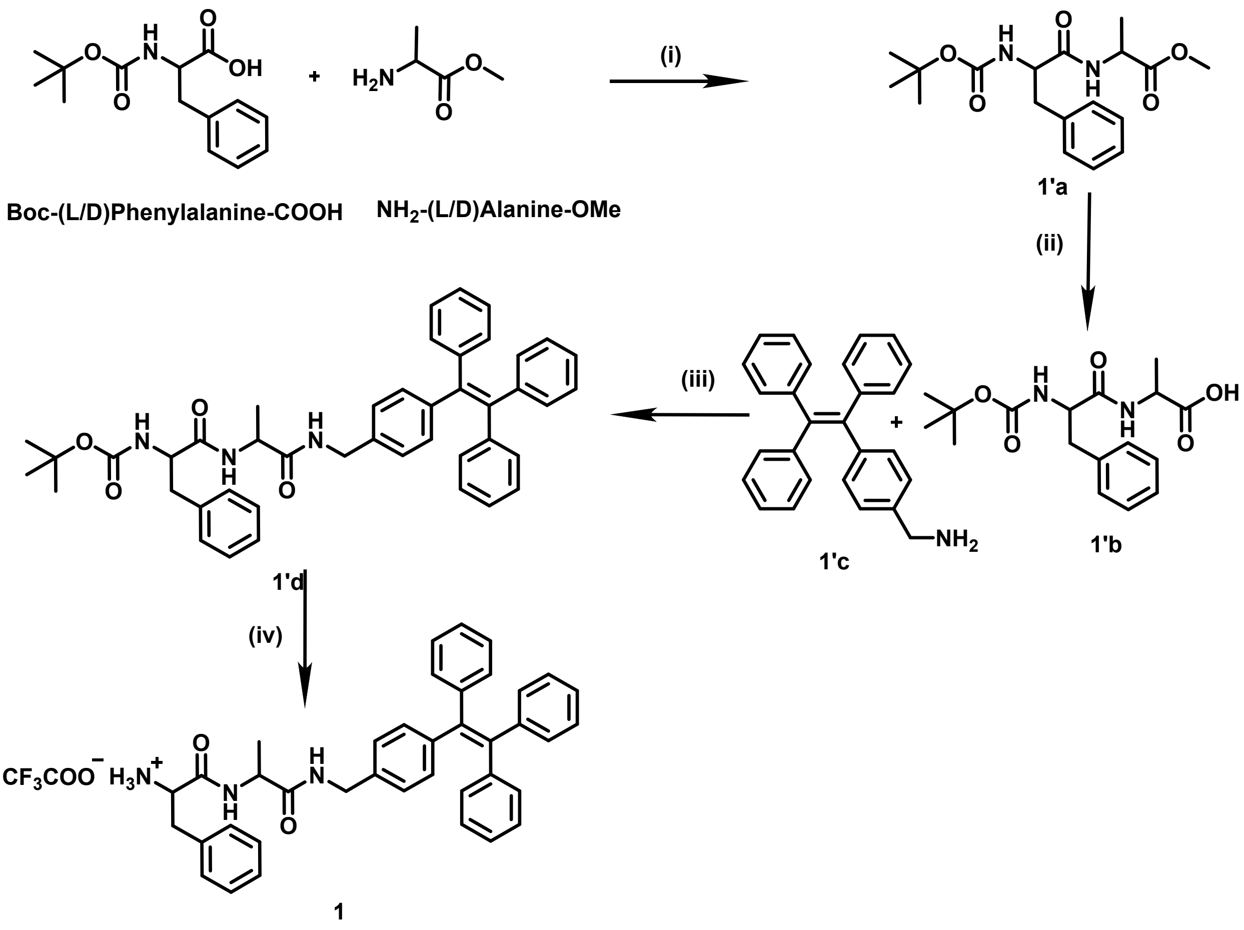

Reagents and Conditions: (i) EDC, DMAP,HOBt,Et_3N, Dry DCM, Room Temperature; (ii) 2N NaOH, EtOH; (iii) EDC, DMAP,HOBt,Et_3N, Dry DCM, Room Temperature; (iv) TFA, Dry DCM

The **compound 1'c** was synthesized according to the reported procedure with minor modifications. After the ascertaining the purity of the compound by ^{1}H-NMR we moved on with synthesis for further functionalization.[4,9]

^{1}H-NMR ($CDCl_3$, 400MHz, δ ppm): 1.61 (s, 2H); 3.78 (s, 2H), 7.10-6.98 (m, 19H).
The molecule TPE-AA (2-(L,L)) was synthesized by following the reported procedure previously by our group.[9]

A total of four FA dipeptides were synthesized by varying the stereogenicity of the amino acids, either with L/D-Phenylalanine or L/D-Alanine. The four peptides are represented as F^{L}-A^{L}. F^{D}-A^{D}, F^{D}-A^{L}, F^{L}-A^{D}. The peptides were synthesized using a common procedure for all the analogues as mentioned above in Scheme-2.[2, 5-7]

Synthesis of Compound 1'a (Boc-Phe-Ala-OMe/ methyl-(tert-butoxycarbonyl) phenylalanylalaninate):

A mixture of Boc-Phenylalanine-COOH (900 mg, 3.39 mmol), NH_2-Alanine-OMe (384 mg, 3.73 mmol), HOBt (458 mg, 3.39 mmol) and DMAP (12.21 mg, 0.33 mmol) were dissolved in 50 mL of dry DCM followed by addition of 500 μL of Et_3N with stirring until a clear transparent solution was obtained. To this solution EDC (1.36 gm, 7.12 mmol) was added and stirred overnight. The resulting mixture was then diluted with 200 mL of DCM and washed 3 times with 1N HCl. This led to phase separation and the organic layer was separated and then passed through anhyd. Na_2SO_4 and filtered. The filtrate was then concentrated in a rotary evaporator to get a crude solid, which was purified by flash column chromatography (60-120 mesh) with $CHCl_3$ as eluting solvent to afford the pure product as a white solid.[2, 8]

^{1}H-NMR ($CDCl_3$, 400MHz, δ ppm): 1.35-1.36 (d, 3H); 1.44 (s, 9H); 3.08-3.11 (m, 2H); 3.74 (s, 3H); 4.37 (br, 1H); 4.53-4.56 (t, 1H); 5.01 (br, 1H); 6.42-6.43 (d, 1H); 7.22-7.34 (m, 5H).
^{13}C-NMR ($CDCl_3$, 100MHz, δ ppm):17.31, 27.42, 37.35, 51.87, 58.81, 79.84, 126.99, 127.94, 129.35, 131.39, 131.48, 157.74, 171.69, 174.72.

Synthesis of Compound 1'b ((tert-butoxycarbonyl)phenylalanylalanine):

To a solution of 1 g (2.58 mmol) of the compound 1'a (methyl-(tert-butoxycarbonyl)phenylalanylalaninate) in 20 mL of EtOH, a solution of 4(N) NaOH (20 mL) was added. The mixture was stirred for 6 h and then acidified with diluted HCl to pH~5-6. The organic solvent was then removed under vacuum and the aqueous residue was then washed with EtOAC (2 x 250 mL). The organic layer was then collected and combined and passed through anhyd. Na_2SO_4. The separated organic layer was then evaporated to get 1'b ((tert-butoxycarbonyl)phenylalanylalanine) as a pure product as judged by TLC.[2]

^{1}H-NMR ($CDCl_3$, 400MHz, δ ppm): 1.23-1.39 (m, 12H); 3.04 (br, 2H); 4.50 (br, 2H); 5.29 (br, 1H); 6.91-6.92 (d, 2H); 7.20-7.29 (m, 5H).

^{13}C-NMR ($CDCl_3$, 100MHz, δ ppm): 17.21 ,26.21 ,36.26 ,51.36,58.86,71.56,127.78, 128.94, 129.31, 131.43, 131.46 ,157.71, 171.73, 174.71,

Synthesis of Compound 1'd (tert-butyl (1-oxo-1-((1-oxo-1-((4-(1,2,2-triphenylvinyl) benzyl)amino)propan-2-yl)amino)-3-phenylpropan-2-yl)carbamate:

Compound 1’b (tert-butoxycarbonyl)phenylalanylalanine) (420 mg, 1.24 mmol), Compound 1’c ((4-(1,2,2-triphenylvinyl)phenyl)methanamine) (361.44 mg, 1.37 mmol), HOBt (167 mg, 1.24 mmol) and DMAP (6 mg, 0.1 mmol) were dissolved in 30 mL of dry DCM followed addition of 200 μL of Et_3N. Then EDC (502.25 mg, 2.62 mmol) was added to the resulting solution and stirred overnight. In the workup step, same procedure was followed as in the synthesis of compound 1’a (methyl-(tert-butoxycarbonyl)phenylalanylalaninate). The crude was purified by column chromatography (60-120 mesh) with mixture of $CHCl_3$ and MeOH (0.5%) as eluting solvent to get a yellowish white solid.[2]

^{1}H-NMR ($CDCl_3$, 400MHz, δ ppm): 1.32(d, 2H), 1.41(s, 12H), 3.20 (d, 2H), 4.32-4.43 (br, 4H), 4.96 (br, 1H); 6.35 (br, 2H); 6.98-7.29 (m, 24H).

^{13}C-NMR ($CDCl_3$, 100MHz, δ ppm): 18.10, 28.38, 38.27, 43.20, 49.17, 126.58, 126.61, 126.84, 127.33, 127.76, 127.79, 127.88, 128.96, 129.36, 131.42, 131.45, 131.72, 136.13, 136.38, 140.62, 141.23, 143.03, 143.80, 171.31, 171.55.

Synthesis of Compound 1/ TPE-AF (1-oxo-1-((1-oxo-1-((4-(1,2,2-triphenylvinyl)benzyl) amino)propan-2-yl)amino)-3-phenylpropan-2-aminium trifluoro acetate):

500 mg (0.86 mmol) of the compound 1’d (tert-butyl (1-oxo-1-((1-oxo-1-((4-(1,2,2-triphenylvinyl)benzyl)amino)propan-2-yl)amino)-3-phenylpropan-2-yl)carbamate) was dissolved in minimum volume of dry DCM. To this 200 μL of TFA was added dropwise and the solution was stirred for 8h. Then the solution was then concentrated under vacuum and washed several times with ether to obtain a yellowish white powder as product.

^{1}H-NMR ($CDCl_3$, 400MHz, δ ppm): 1.26-1.28 (d, 3H); 3.11-3.16 (br, 2H); 4.27-4.48 (m, 4H); 6.17 (br); 6.92-7.21 (m, 25H); 7.88-7.89 (d,1H).

^{13}C-NMR ($CDCl_3$, 100MHz, δ ppm):17.92, 37.50, 43.60, 49.97, 55.25, 126.68, 126.74, 127.81, 127.86, 127.88, 128.38, 129.35, 129.41, 131.37, 131.87, 133.29, 134.74, 140.37, 141.63, 143.64, 143.73, 173.06.

FT-IR (cm^{-1}): 3280.43, 3063.55, 2926.26, 2857.72, 1656.63, 1530.09, 1444.05, 1202.61. 1140.09.

ESI-MS: Calculated Mass- 580.30 $[M]^+$; 581.30 $[M+H]^+$; 603.3 $[M+Na]^+$, Mass obtained-580.1378 $[M]^+$, 581.1398 $[M+H]^+$ and 603.1160 $[M+Na]^+$.

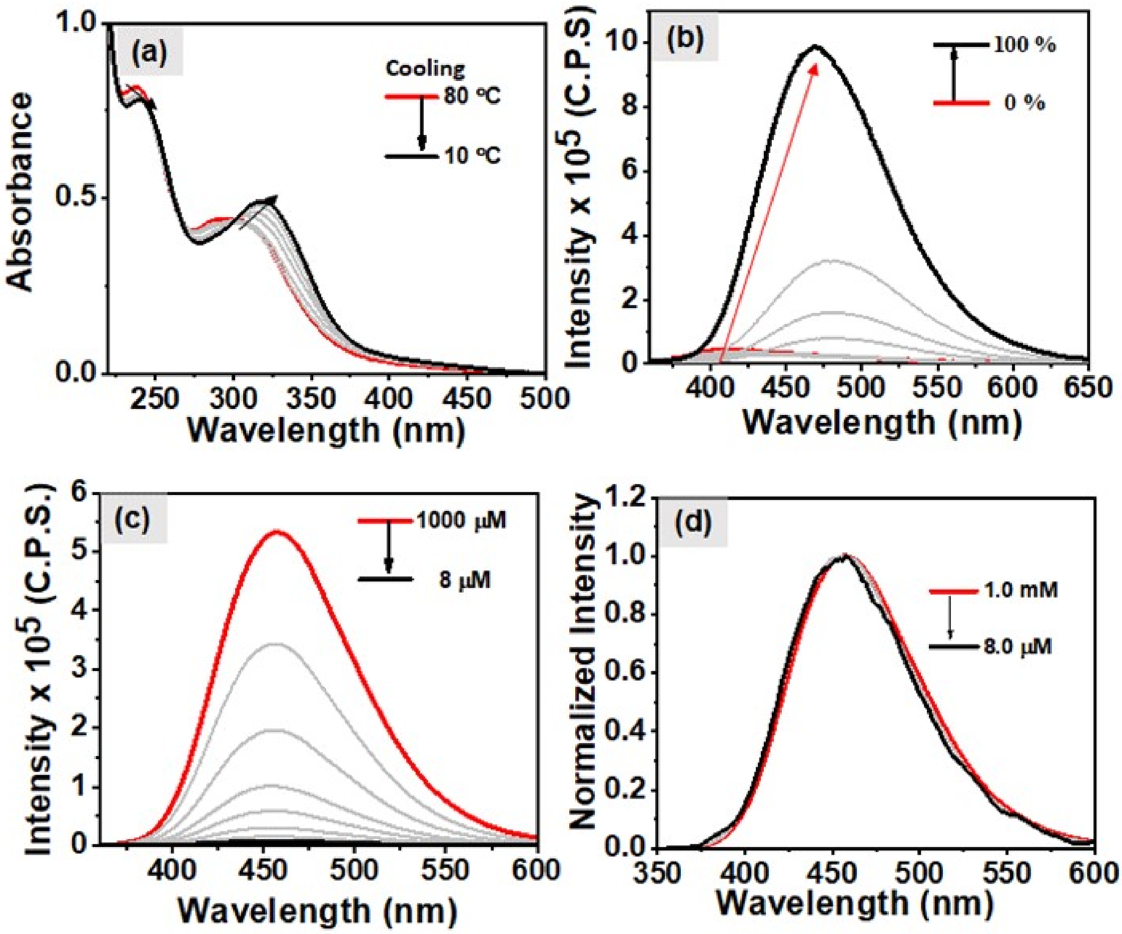

Figure S1: a) UV-Vis spectra of **1**-(L, D) during cooling process, b) Fluorescence Spectra of the **1**-(L, D) with varying concentration of tetrahydrofuran & water, c) Concentration dependent Fluorescence Spectra and d) Normalized Fluorescence spectra at different concentration of the 1-(L, D) molecule in water.

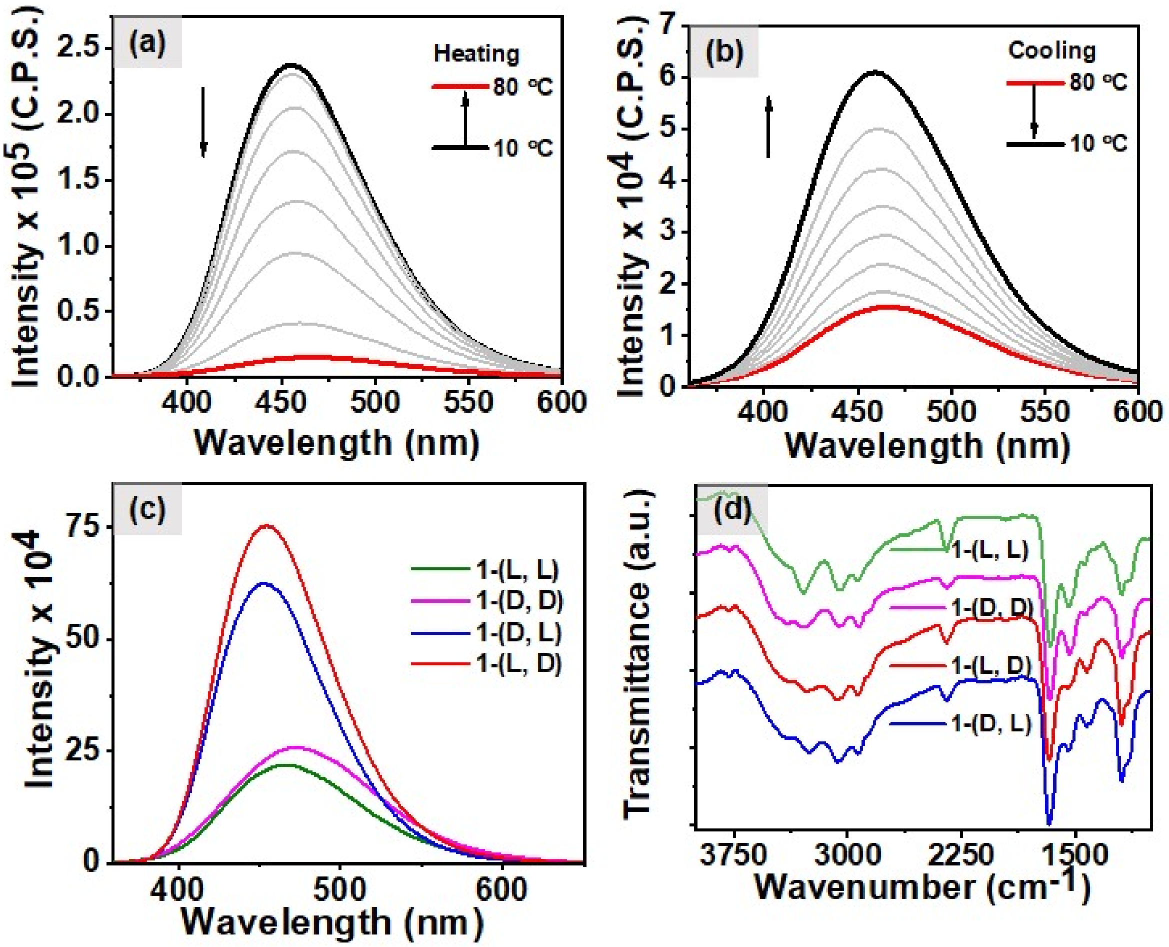

Figure S2: Temperature dependent Fluorescence Spectra of the compound 1-(L, D) a) During heating and b) Cooling process. .c) Fluorescence spectra of the different isomers in aqueous media d) The FT-IR spectra of the assembled molecules.

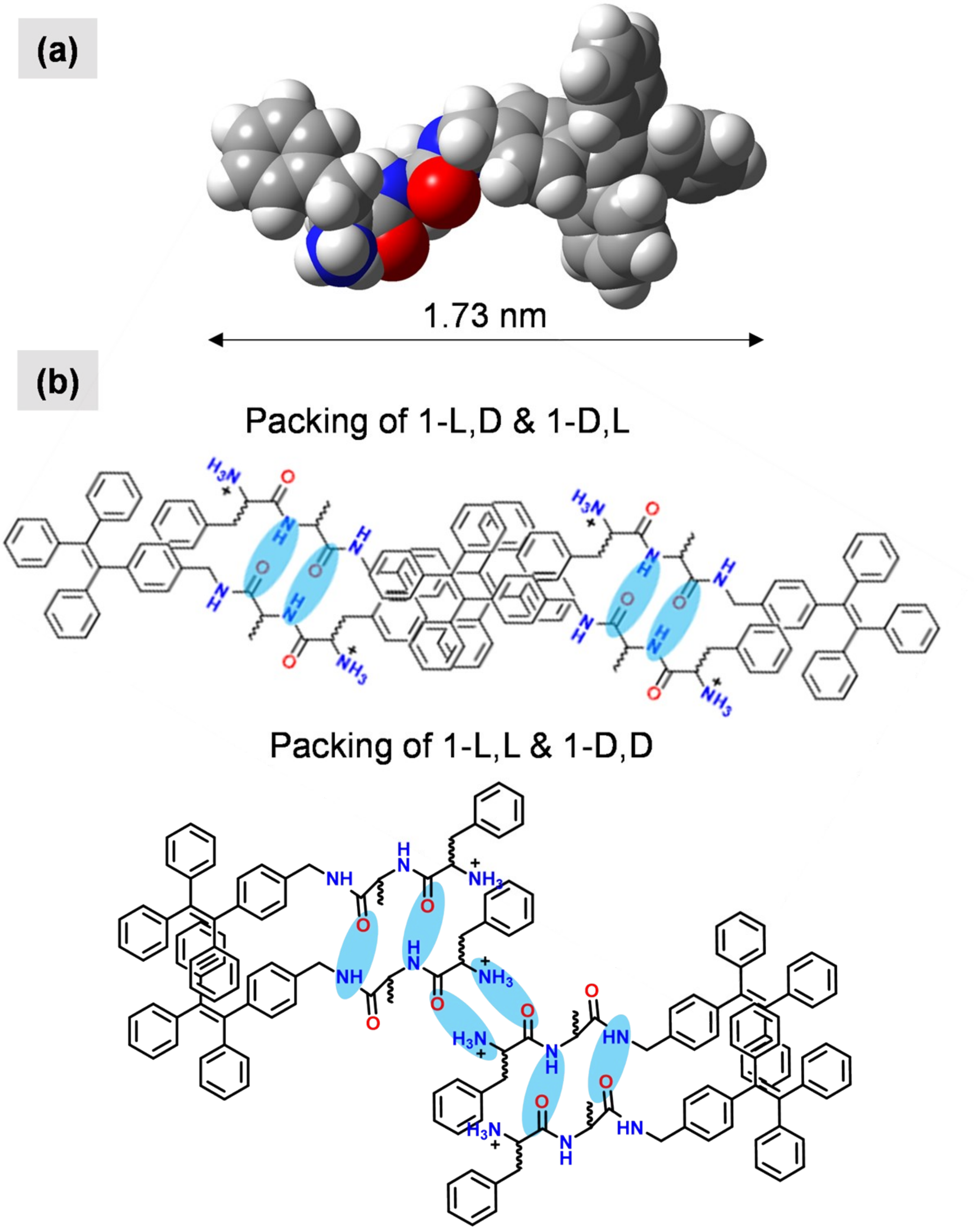

Figure S3: a) The Gaussian minimized structure of the 1-(L,L) in CPK model showing the molecular distance. b) The proposed molecular packing and H-Bonding pattern of the homochiral and the heterochiral system based on SAXRD diffraction.

Molecular structure Optimization The energy-minimized structures obtained using DFT for the heterochiral and homochiral systems. In the heterochiral system, the carbonyl groups are oriented in opposite directions, whereas in the homochiral system, they are oriented in the same direction. These preferred orientations of the carbonyl groups lead to the formation of different hydrogen bonds, as illustrated in Figure S3, which supports the observations from the CD spectra, where heterochiral systems tend to form β-sheet like structures, while homochiral systems are inclined to form helices.

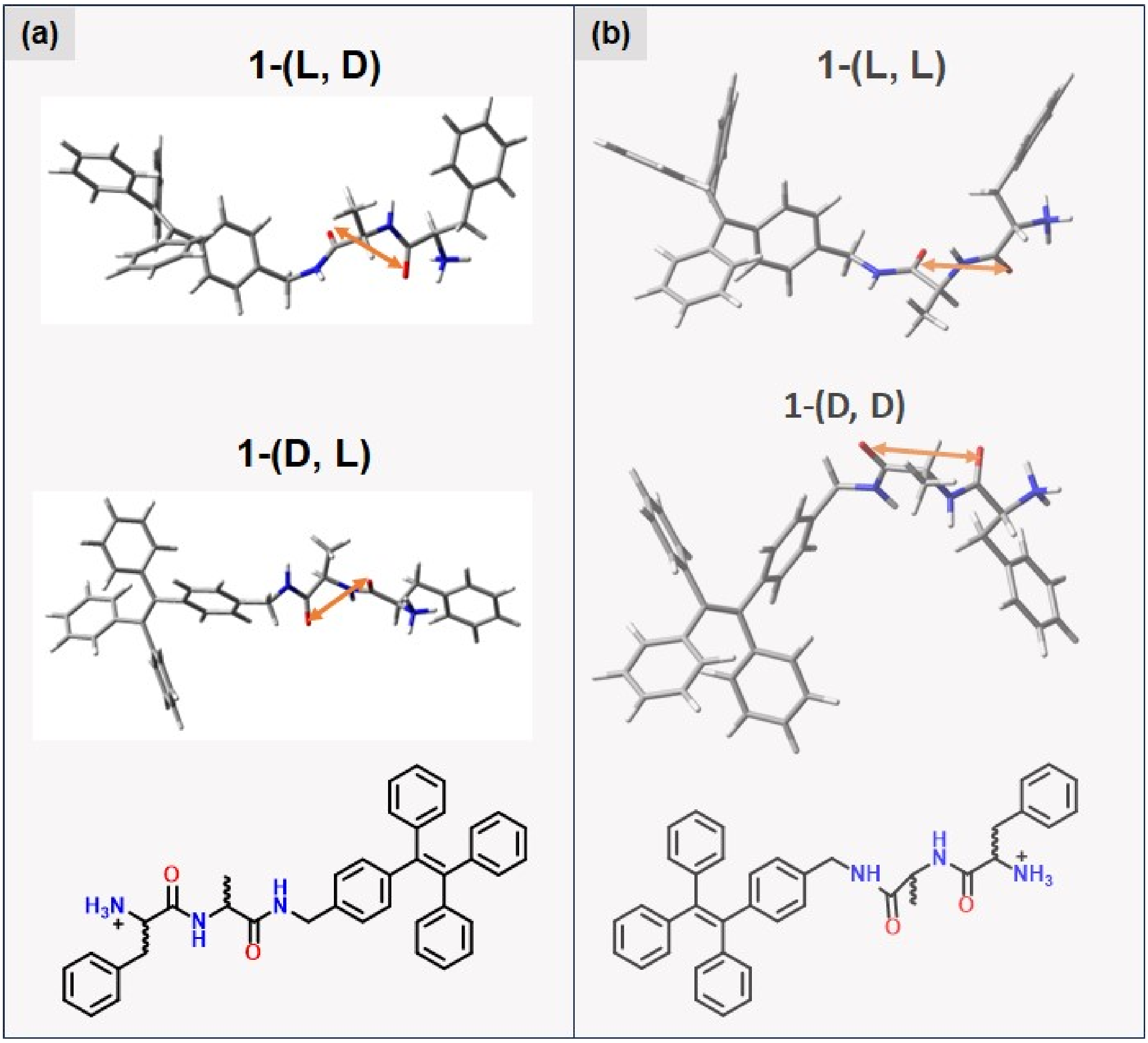

Figure S4: The energy minimized structure by DFT in ball stick model of the a) heterochiral compounds & b) homochiral chiral molecules.

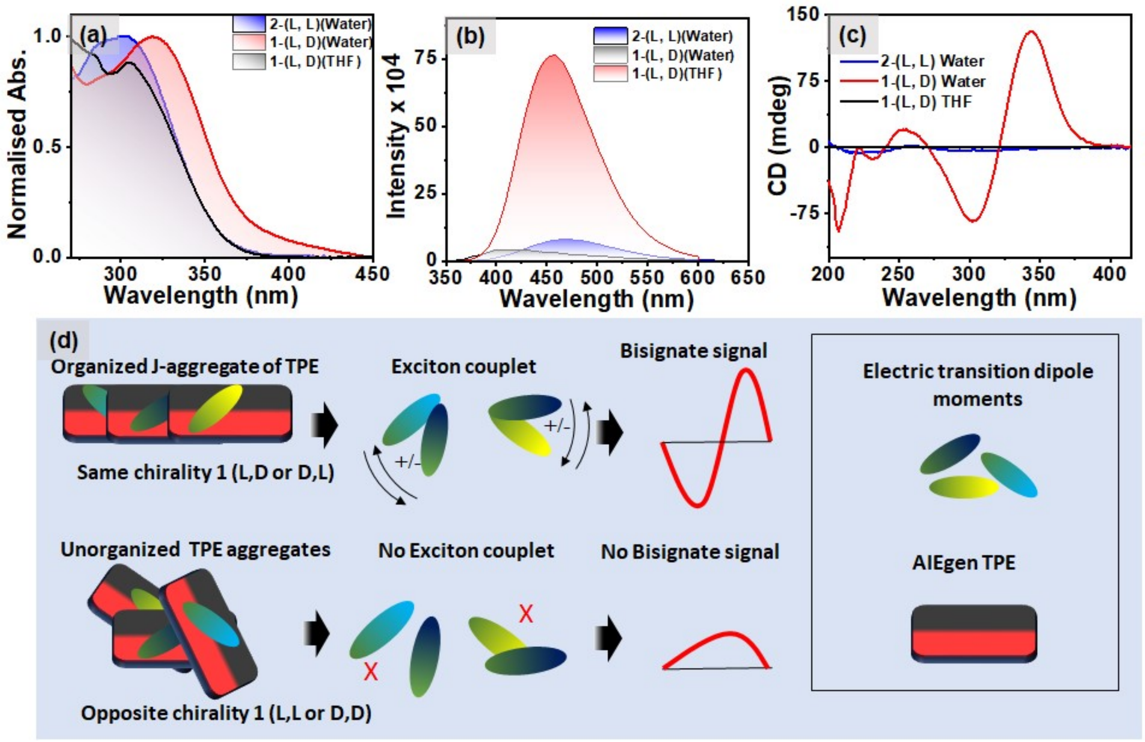

Figure S5: a) Normalized UV-Vis spectrum, b) Fluorescence spectrum and c) Circular dichroism spectrum of the molecule 2-(L, L) compared with the aggregated TPE state formed by 1-(L, D). d) Schematic illustration demonstrating the origin of bisignate CD signal due to exciton coupling in the heterochiral systems and absence of bisignate signal in homochiral system

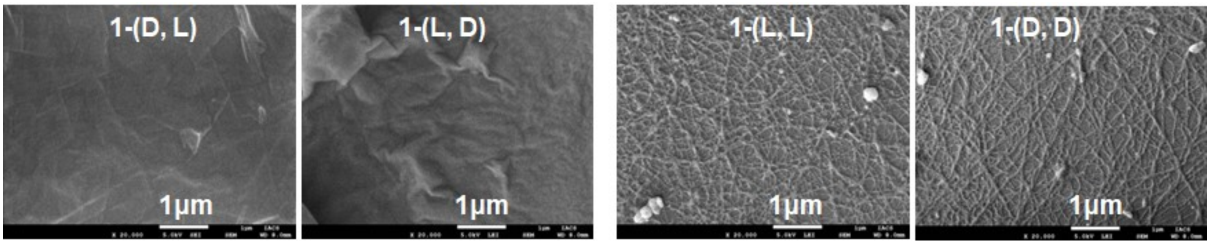

Figure S6: SEM images of the 1-(L, D) and 1-(D, L) system aggregates and 1-(L, L) & 1-(D, D) system aggregates.

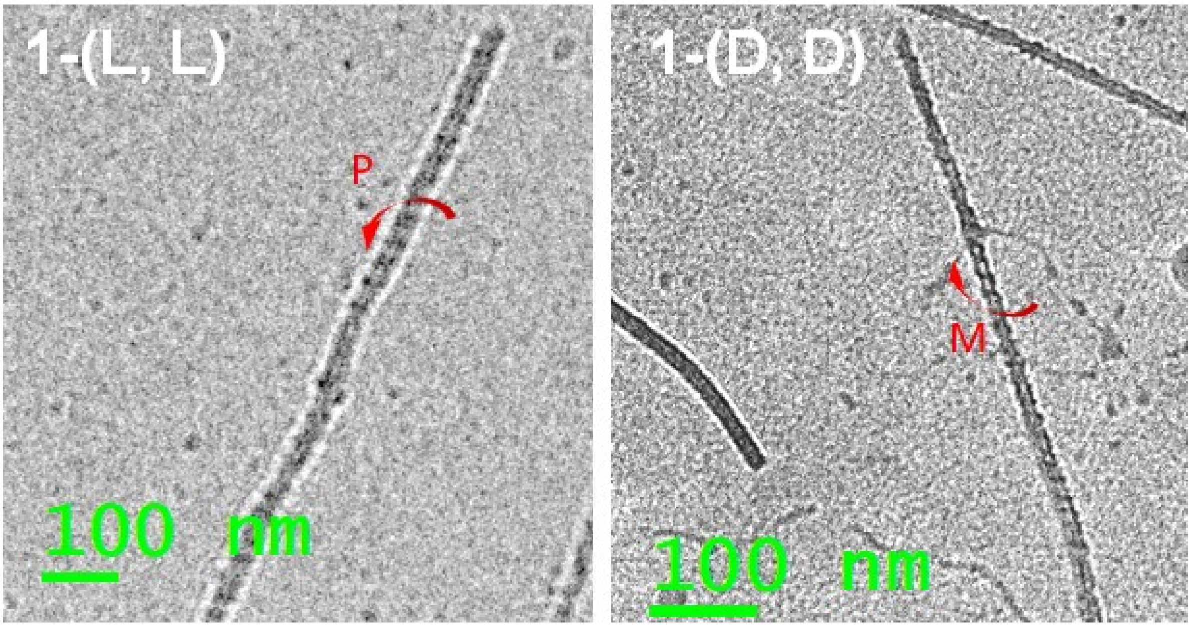

Figure S7: The cryo-EM images showing the handedness of helix formed by the assemblies of the homochiral molecules.

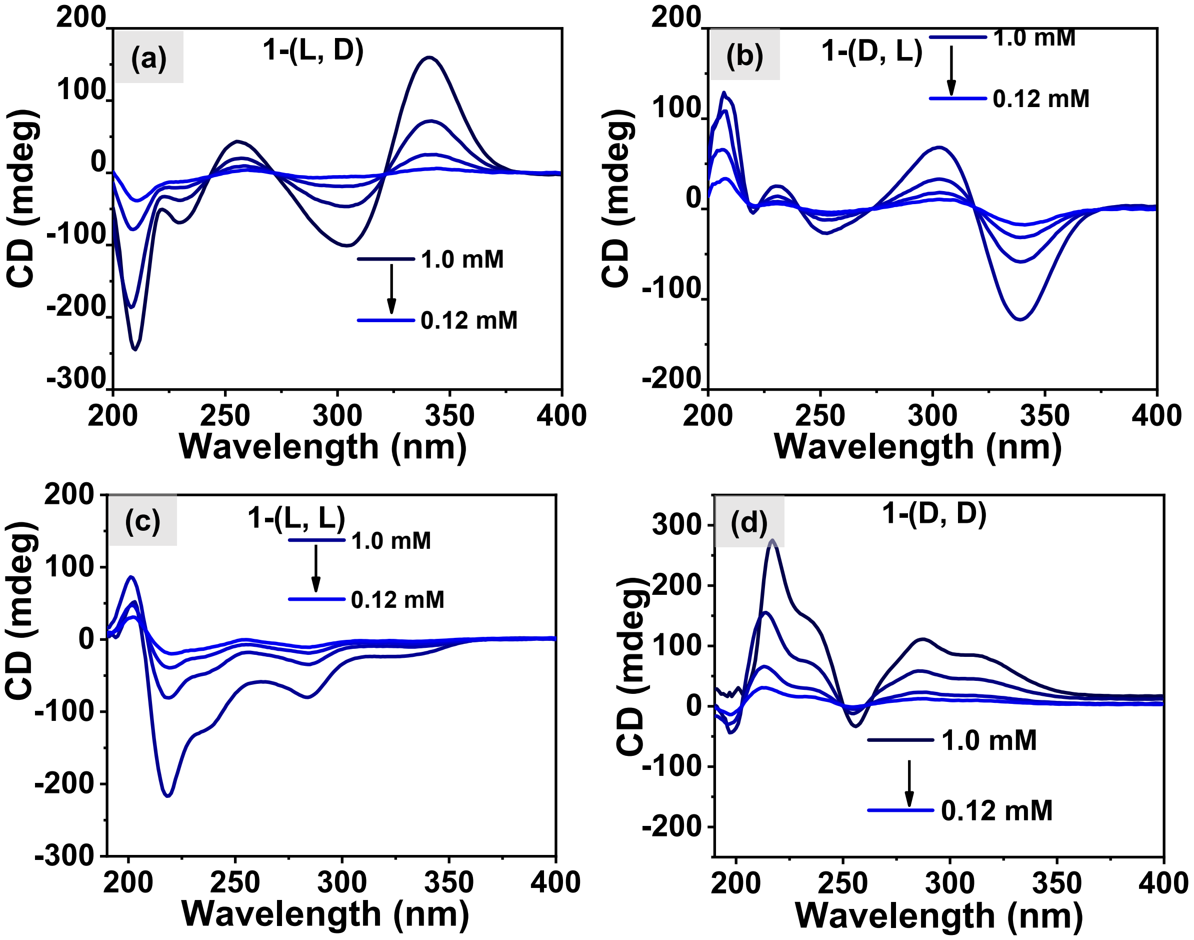

Figure S8: Concentration-dependent circular dichroism spectra of the homochiral (a, b) and heterochiral (c, d) dipeptides in water.

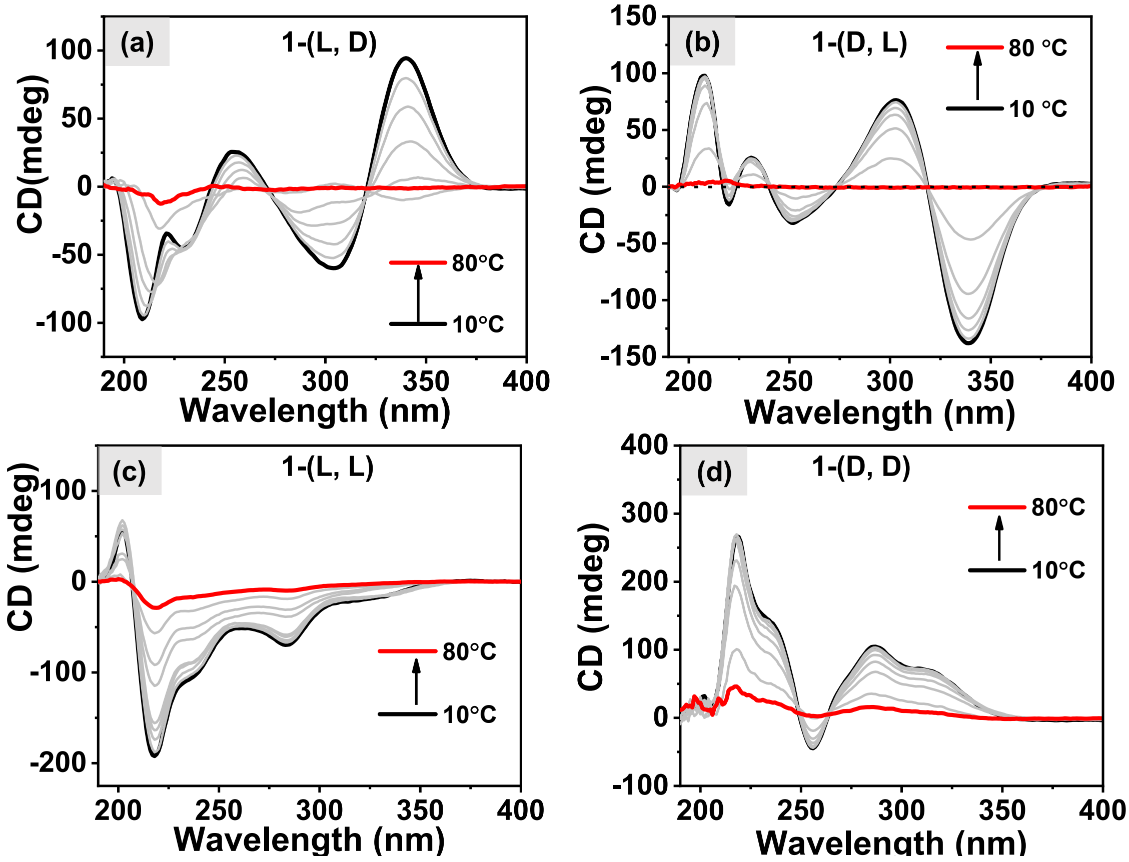

Figure S9: The variations observed in the CD spectra of the heterochiral systems (a and b) and homochiral systems (c and d) during heating of the aqueous solutions of different molecules.

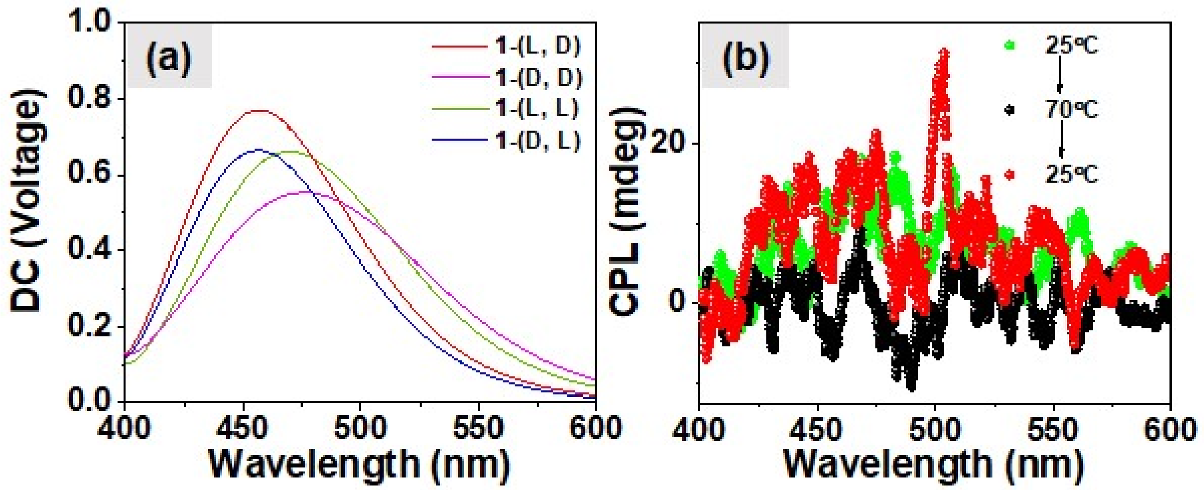

Figure S10: a) The corresponding DC voltages measured during the CPL measurements of the dipeptides with different stereochemistry, and b) Variable temperature CPL spectra of the system 1-(D, D).

Table S1. Comparison of different circularly polarized luminescent materials with reported g_{lum} values.

Sl. No.	System Description	Method	g_{lum}	References
1.	**Aggregates of heterochiral and homochiral peptide-TPE conjugates**	**Water**	**Heterochiral Sheets [7.5 (± 0.04) x 10^{-3}]**<br>**Homochiral Fibrils [1.3 (± 0.05) x 10^{-3}]**	***This Work***
2.	Morphology controlled Circular polarized Luminescence from chiral small molecule	Water & THF mixture	10^{-4} (0D nanospheres)<br>10^{-3} (2D flake)<br>10^{-2} (3D nanoflakes)	*Chem. Sci.*, **2019**, 10, 6821–6827
3.	Tetraphenyl ethylene attached to amino acid, L-Leucine (methyl ester)	DCE and Hexane Mixture	0.02	*J. Mater. Chem. C.* **2015**, 3, 2399-2404
4.	Helical Sulfono-γ-AA peptides that manifest Aggregation-Induced Emission in water	Water (Polymer)	0.012	*J. Am. Chem. Soc.* **2019**, 141, 12697-12706
5.	Pyrene-appended glucono gelators	Ethanol and water solution	2.5 x 10^{-3}	*RSC Adv.*, **2020**, 10, 6772–6776
6.	Symmetrical amino acid (L/D-glutamic acid) appended Naphthalene Di-imide derivative	Methanol and Trifluoro acetic acid	~10^{-2} (nano-bamboo) ~10^{-3} (nanosheets)	*Angew. Chem. Int. Ed.* **2021**, 60, 16615–16621
7.	Cis and gem-TPE Diquaternary Ammonium complexes with DNA	Drop-cast film	0.0028	*Org. Lett.* **2020**, 22, 1836−1840
8.	A chiral AIEgen based on silole core with chiral sugar pendants and chiral perturbative moiety	Hexane and DCM suspension	0.32	*Chem. Sci.* **2012**, 3, 27372747
9.	Emissive Charge transfer complex based on Pyrene & tetracyanobenzene/	Charge transfer complex	0.017 (in film)<br>0.017 (in gel casted Film)	*Angew. Chem.* **2019**, 131, 7087-

	tetracyanoethylene/ tetra-cyanoquinodimethane	formed by crystallization, grinding and gelation		7093
10.	Chiral Supramolecular polymer	*n*-Decane and THF mixture	4.6 x 10^{-3}	*Chem. Eur. J.* **2020**,26,12874–12881
11.	Helically chiral 1,1′-bitriphenylenes	Chloroform Solution	-0.03	*J. Am. Chem. Soc.* **2012**, 134, 4080-4083
12.	(S)-(−)-1-(1-Naphthyl) ethylamine ((S)-NEA) and (R)-(+)-1-(1-naphthyl) ethylamine ((R)-NEA) functionalized perovskites	Flim cast from DMSO mixture	3 × $10^{−3}$	*ACS Nano* **2020**, 14, 6, 7610–7616

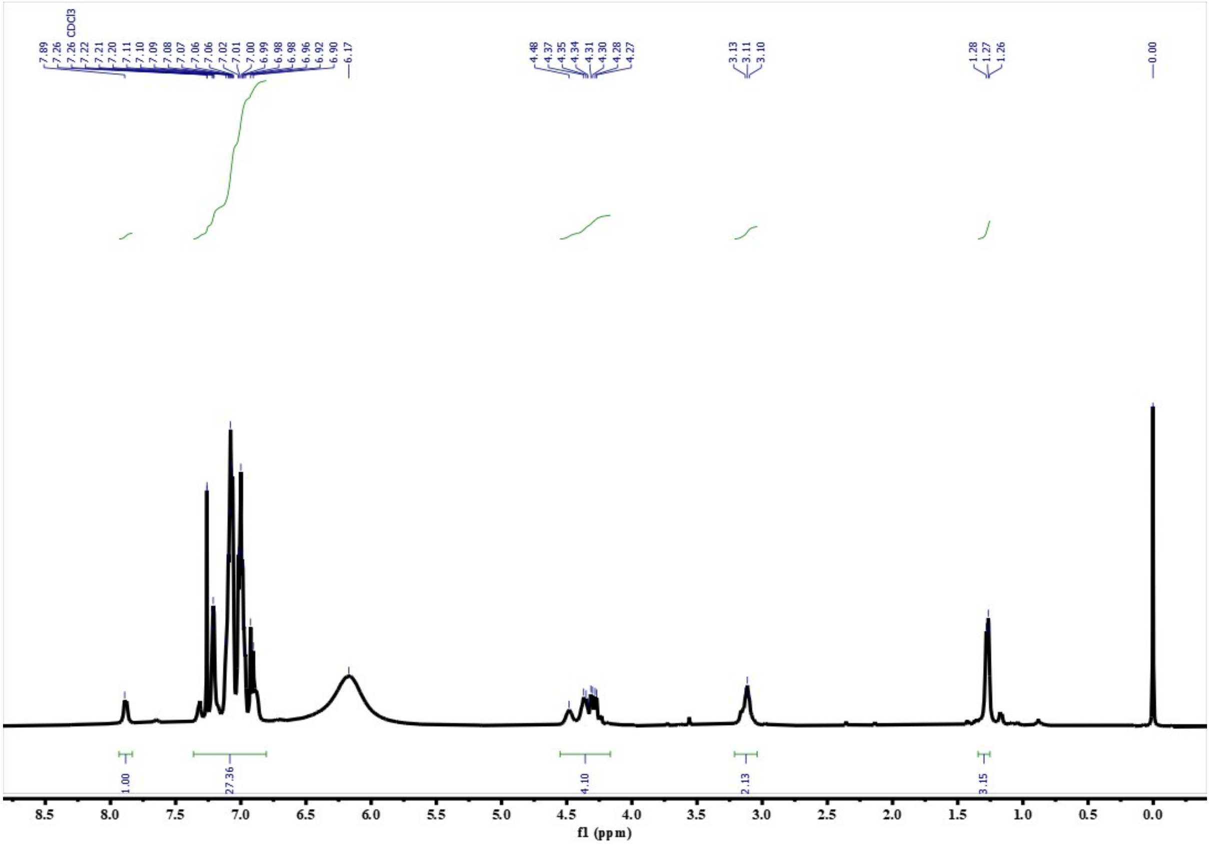

Figure S11: ^{1}H NMR spectra of the **TPE-AF (1) / TPE-A^{L}-F^{L}** in $CDCl_3$.

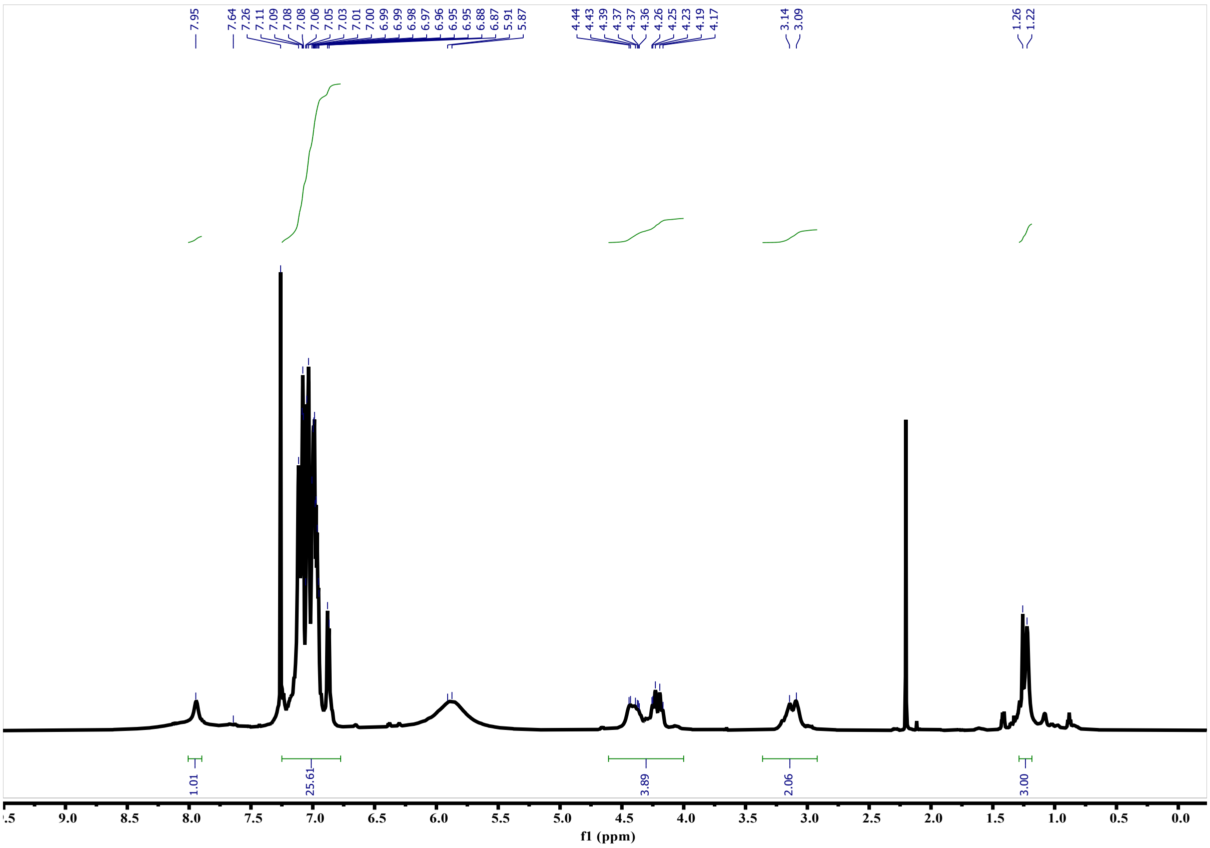

Figure S12: ^{1}H NMR spectra of the **TPE-AF (1) / TPE-A^{D}-F^{D}** in $CDCl_3$.

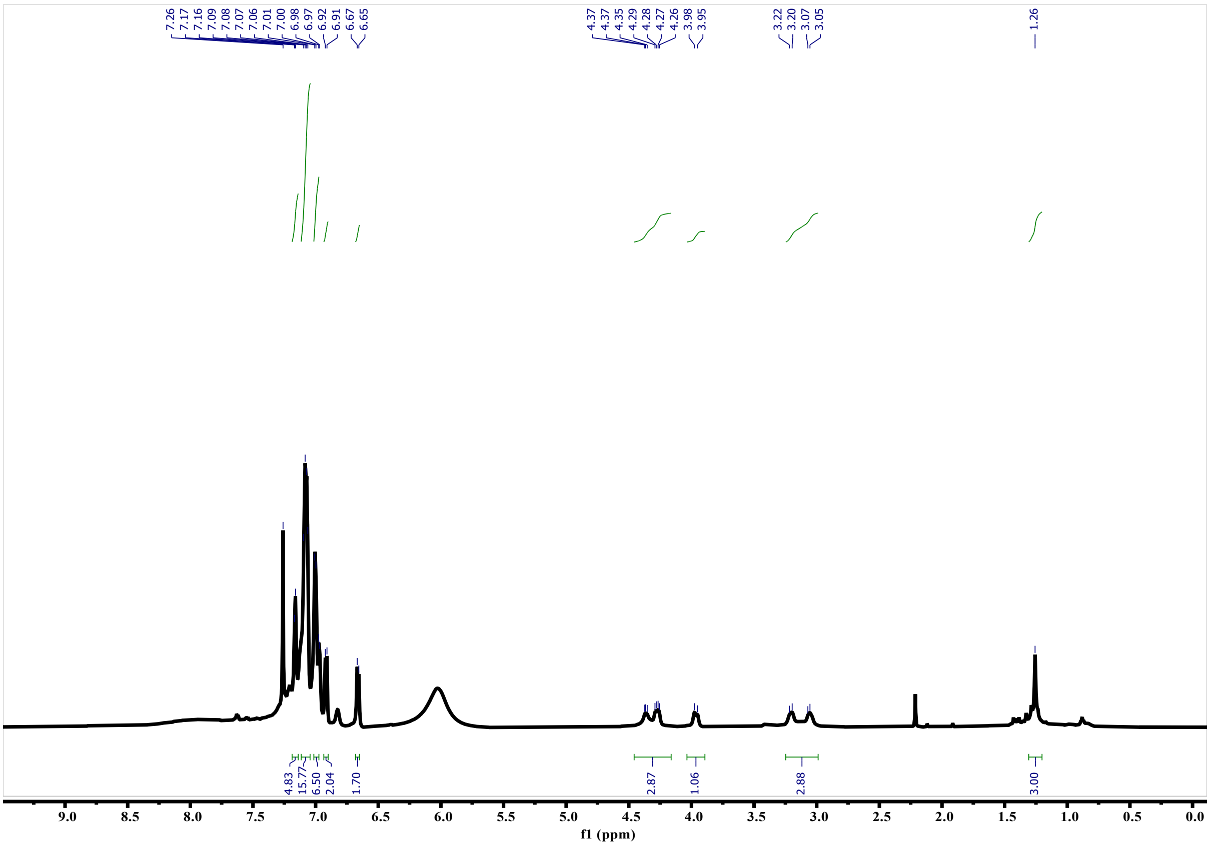

Figure S13: ^{1}H NMR spectra of the **TPE-AF (1) / TPE-A^D-F^L** in $CDCl_3$.

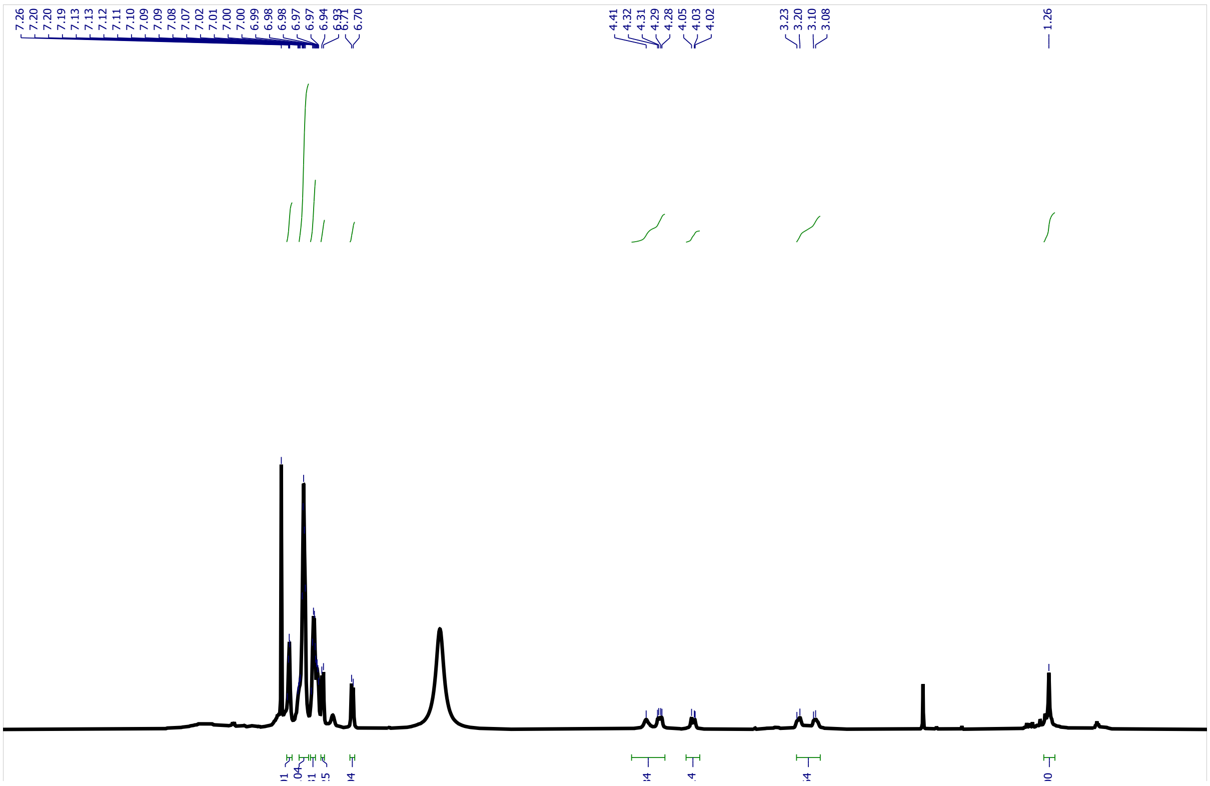

Figure S14: ^{1}H NMR spectra of the **TPE-AF (1) / TPE-A^D-F^L** in $CDCl_3$.

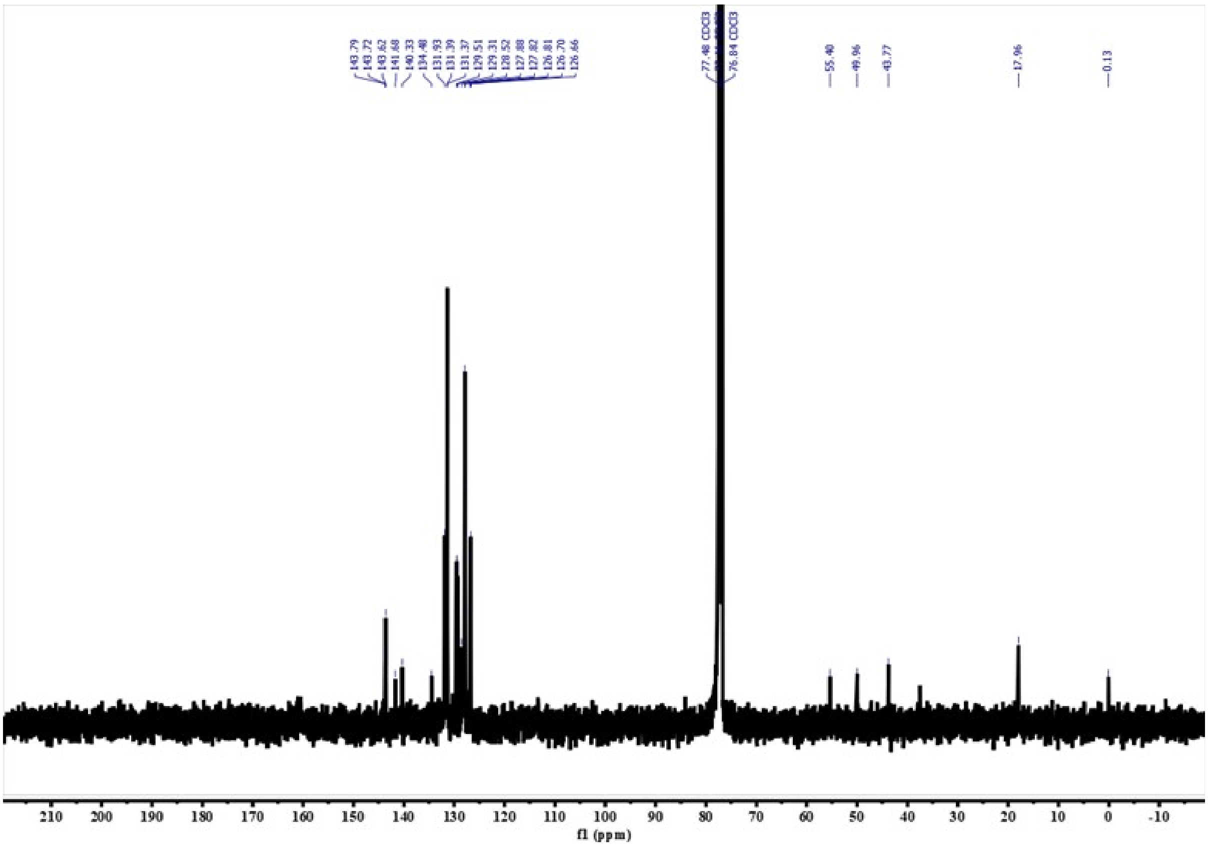

Figure S15: ^{13}C NMR spectra of **TPE-AF (1)/TPE-A^{L}-F^{L}** in $CDCl_3$

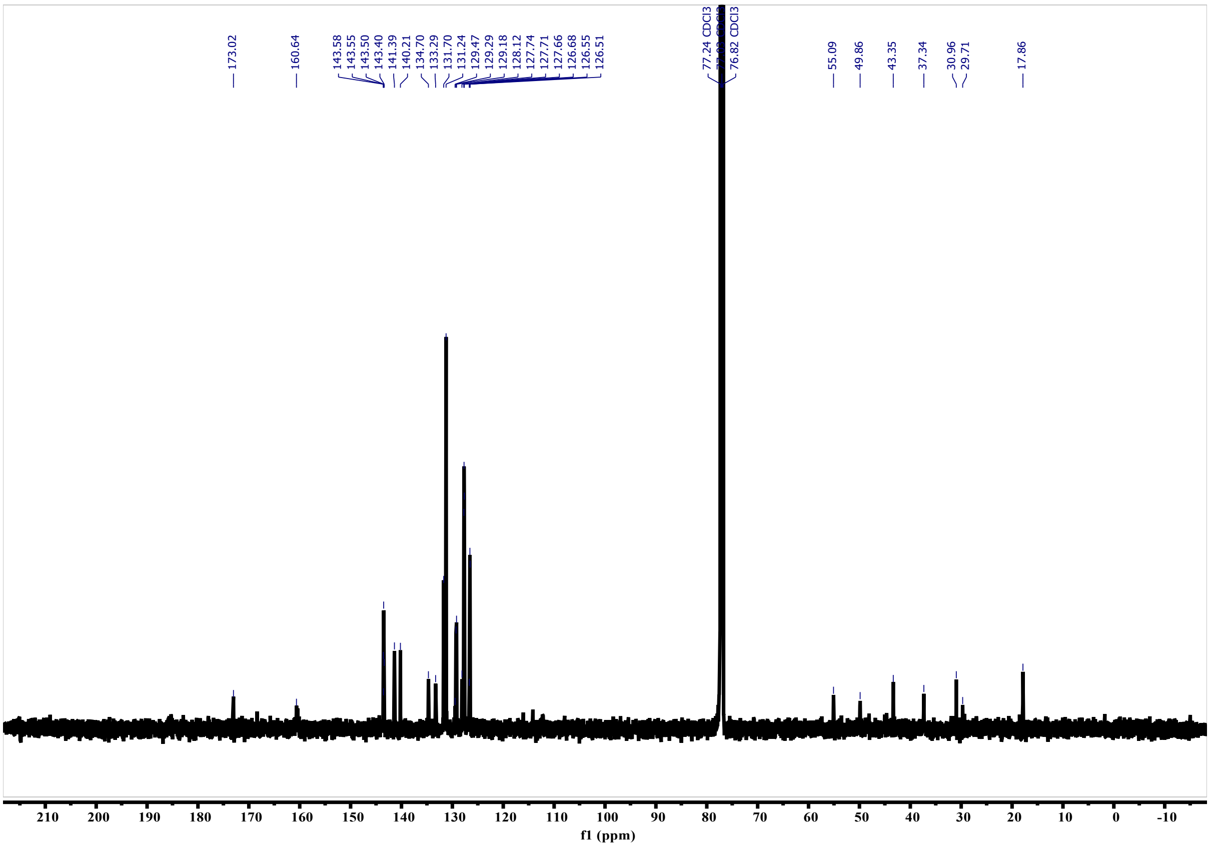

Figure S16: ^{13}C NMR spectra of **TPE-AF (1)/TPE-A^{D}-F^{D}** in $CDCl_3$

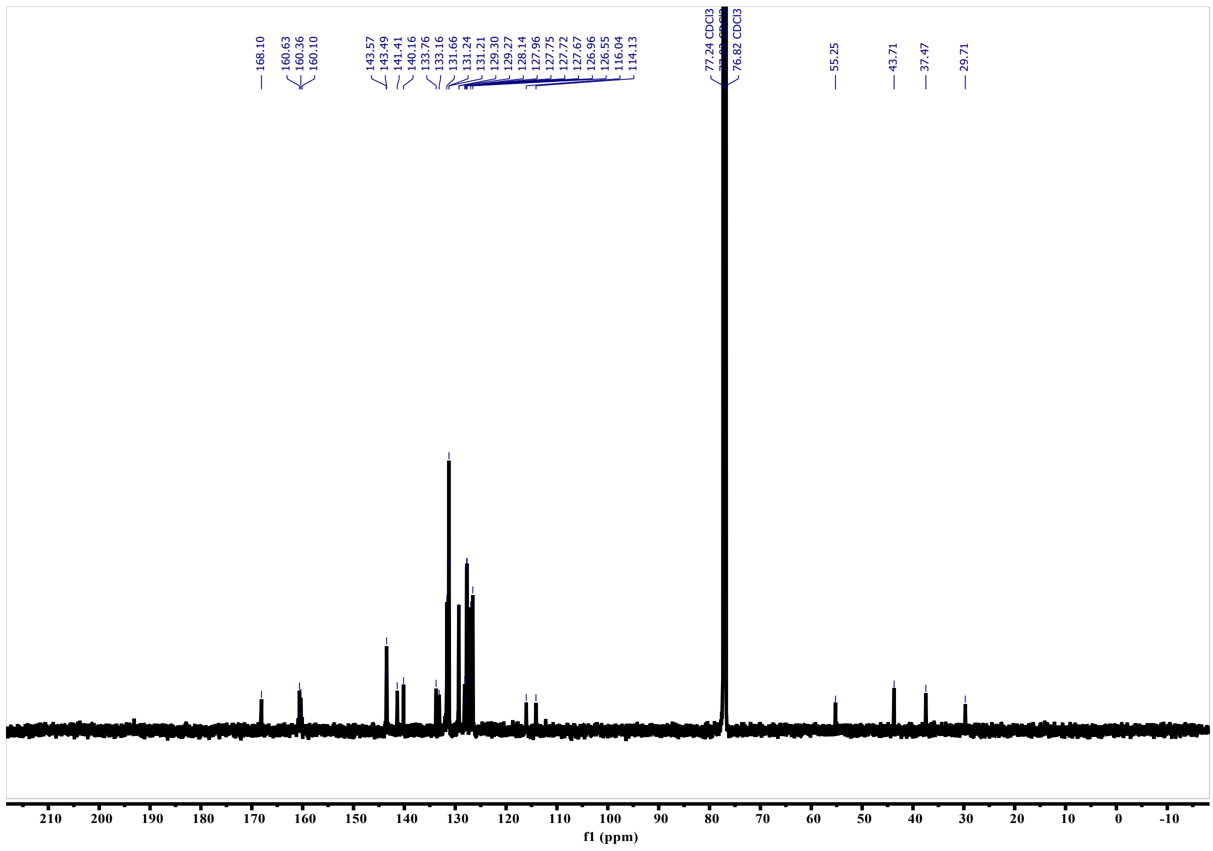

Figure S17: ^{13}C NMR spectra of **TPE-AF (1)/TPE-A^{D}-F^{L}** in $CDCl_3$

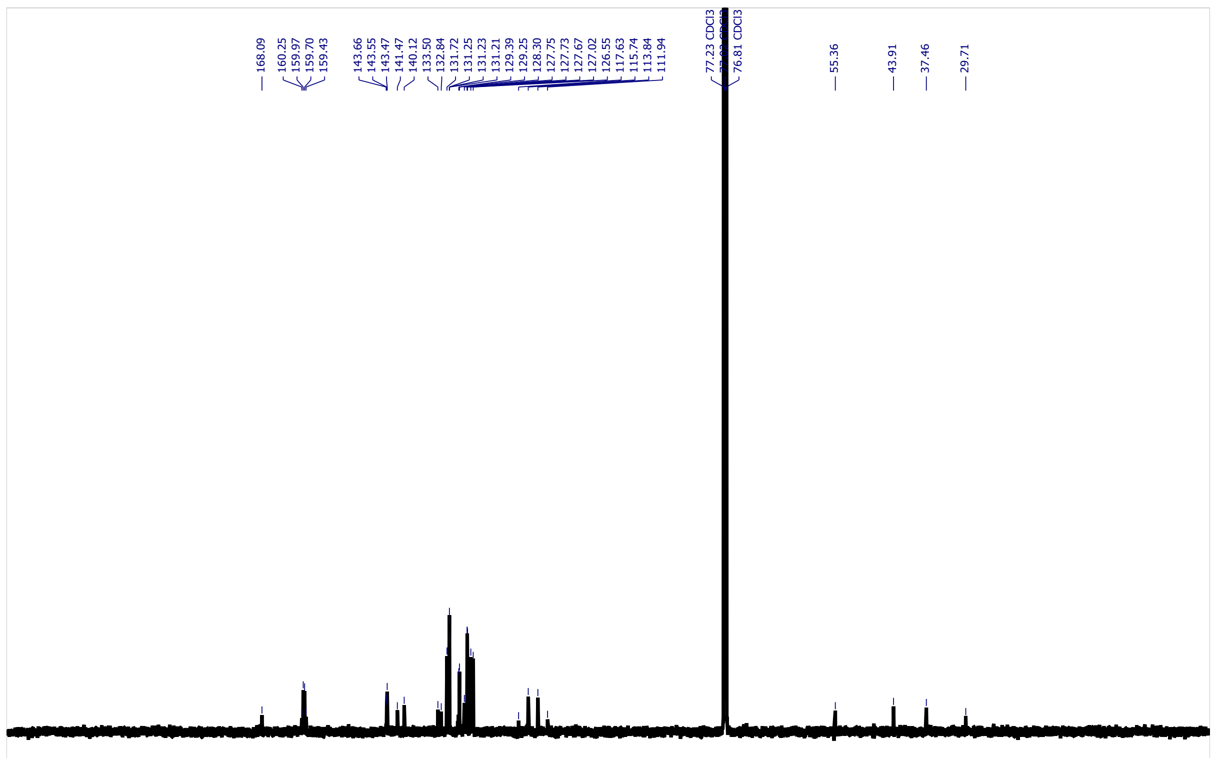

Figure S18: ^{13}C NMR spectra of **TPE-AF (1)/TPE-A^{L}-F^{D}** in $CDCl_3$

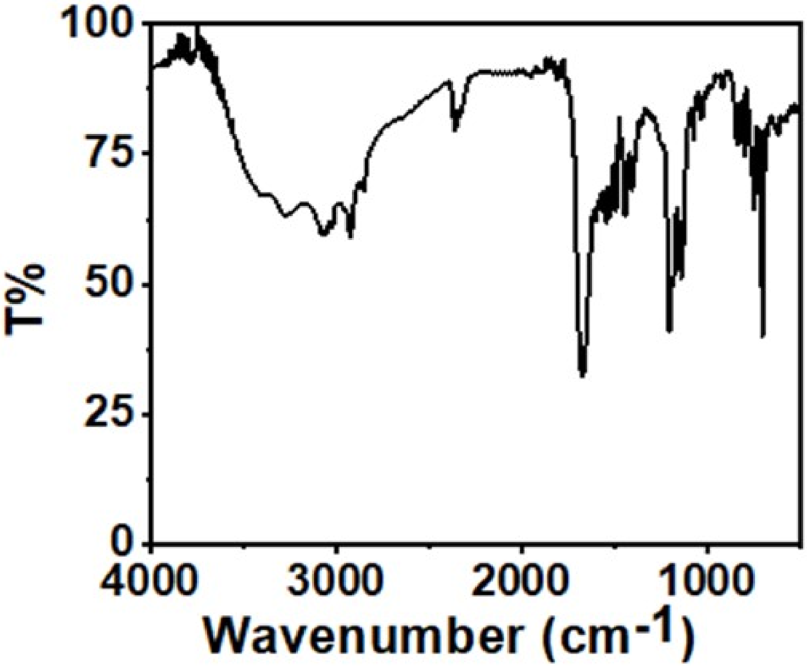

Figure S19: FT-IR spectra of **TPE-AF (1)**.

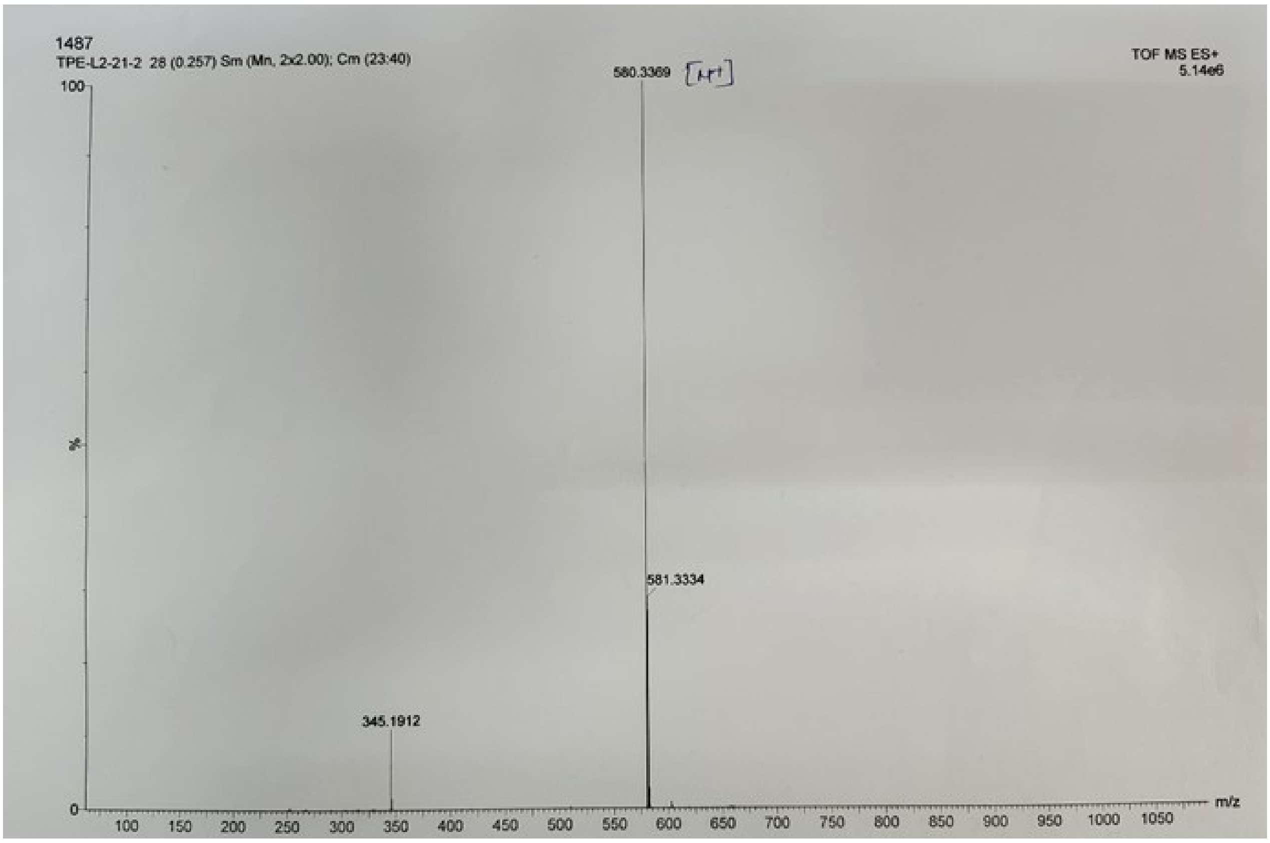

Figure S20: ESI-MS spectra **TPE-AF (1)/TPE-A^{L}-F^{L}.**

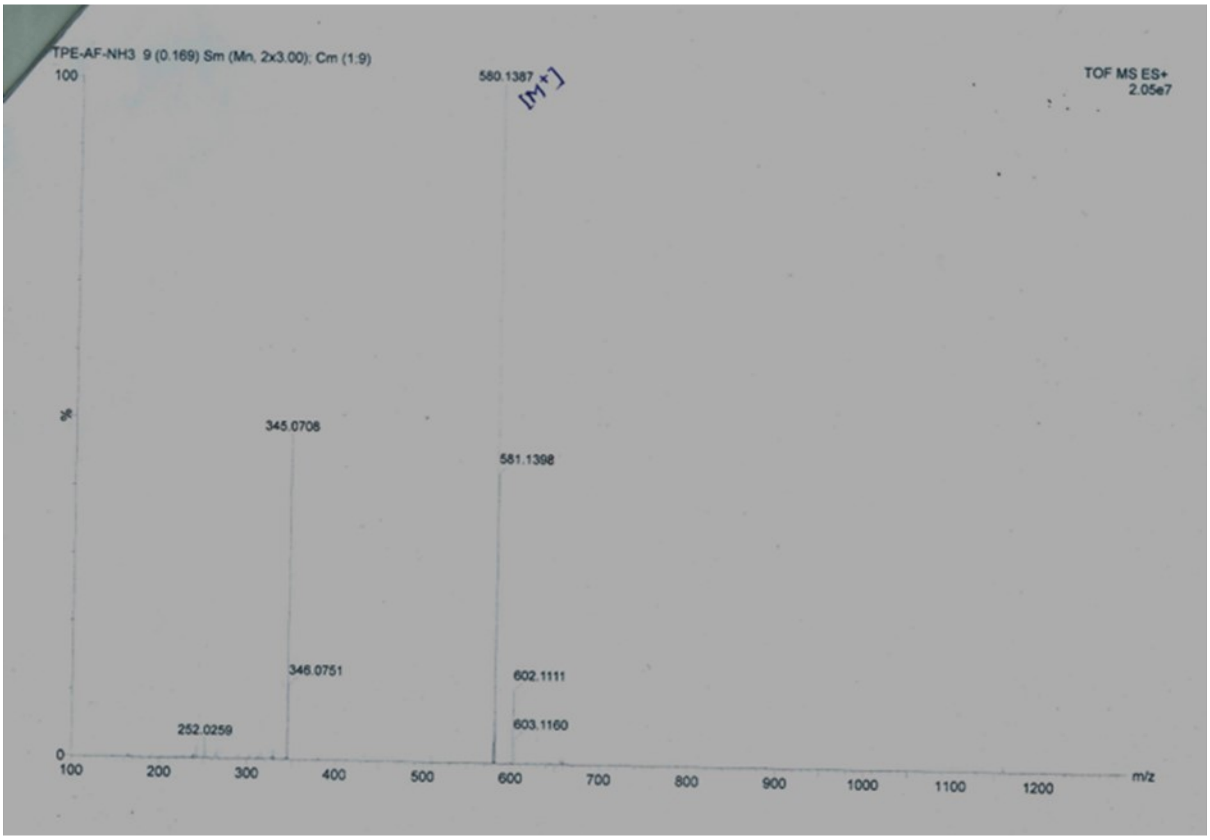

Figure S21: ESI-MS spectra **TPE-AF (1)/TPE-A^{D}-F^{D}.**

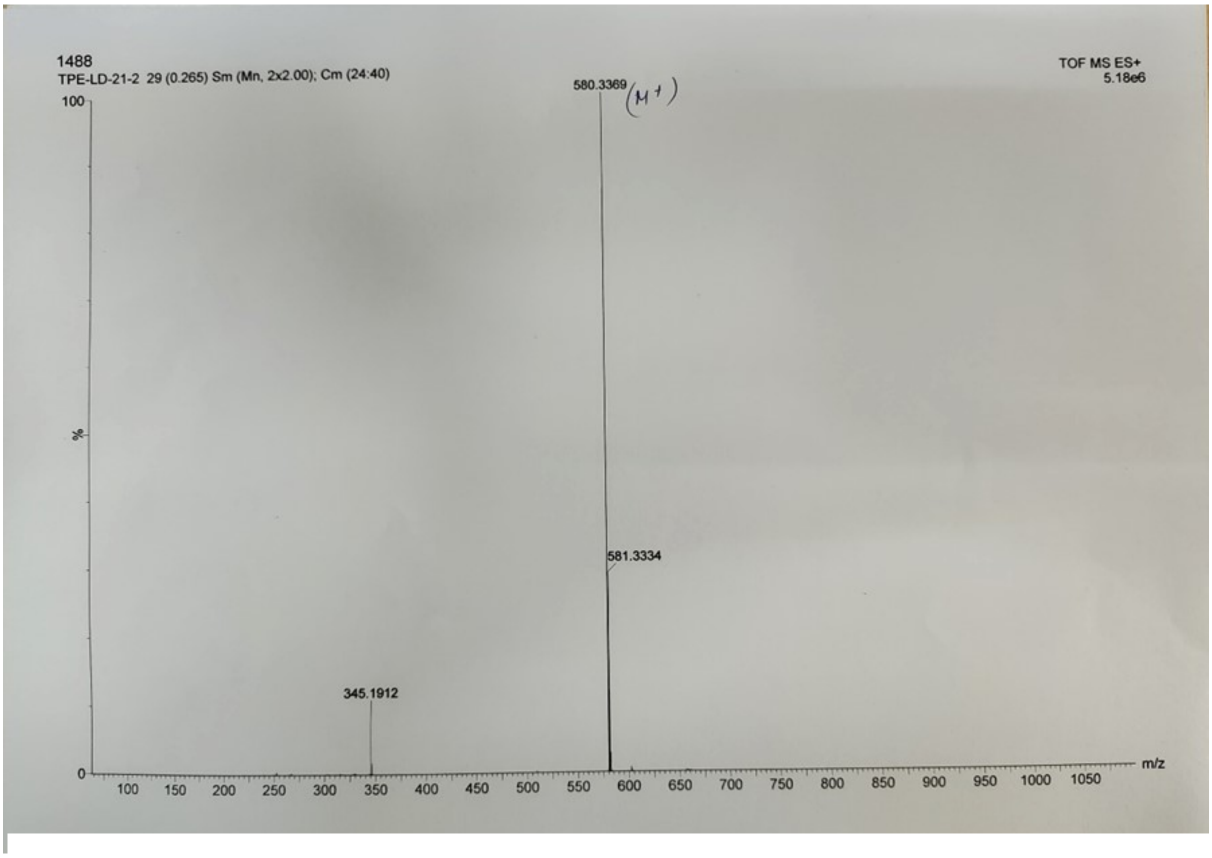

Figure S22: ESI-MS spectra **TPE-AF (1)/TPE-A^{L}-F^{D}.**

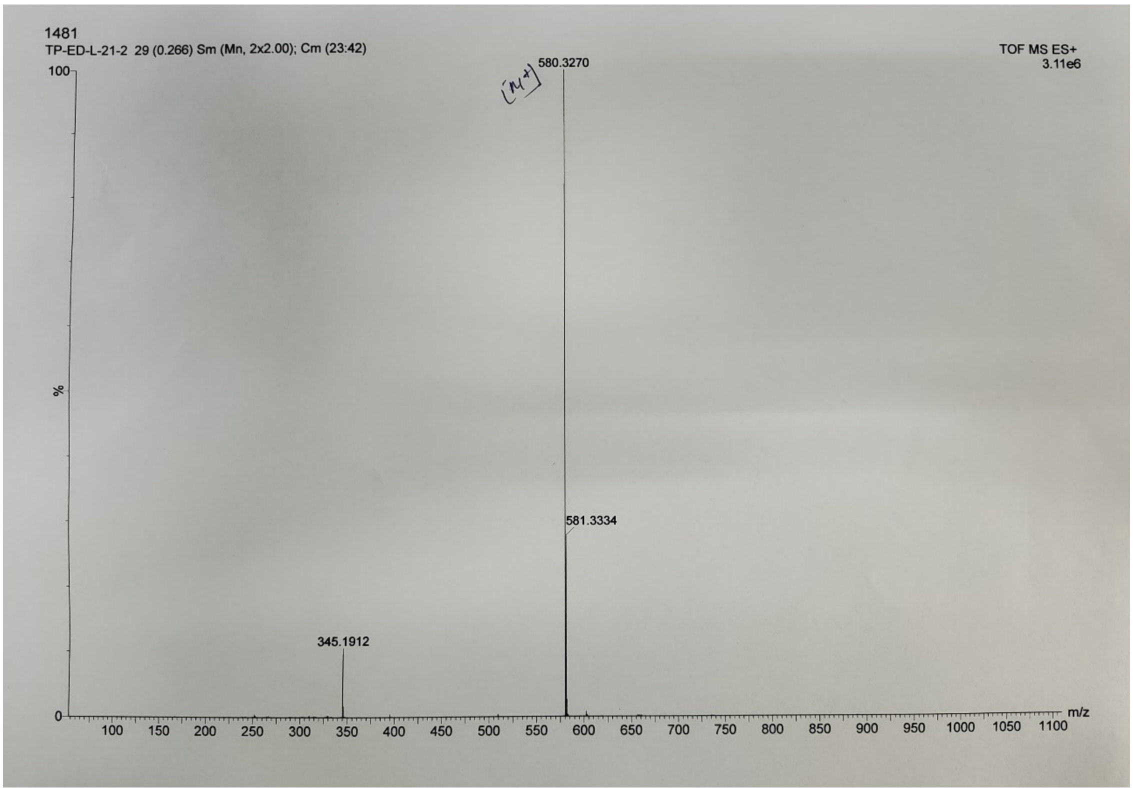

Figure S23: ESI-MS spectra **TPE-AF (1)/TPE-A^{D}-F^{L}.**

1. N. O. Thiel, B. Kaewmee, T. Tran Ngoc and J. F. Teichert, A Simple Nickel Catalyst Enabling an E-Selective Alkyne Semihydrogenation, *Chem, Eur. J.l*, 2020, **26**, 1597-1603.
2. Umesh, S. Bera and S. Bhattacharya, Dual Circularly Polarized Luminescence (CPL) and Piezoelectric Responses in Self-Assembled Chiral Nanostructures Derived from a Dipeptide Based Piezorganogel, *Small*, **2023**, 2308104.
3. D. Quiroga, L. D. Becerra and E. Coy-Barrera, Ultrasound-Assisted Synthesis, Antifungal Activity against Fusarium oxysporum, and Three-Dimensional Quantitative Structure–Activity Relationship of N,S-Dialkyl Dithiocarbamates Derived from 2-Amino Acids, *ACS Omega*, 2019, **4**, 13710-13720.
4. Y. Zhao, Y. Wu, G. Yan and K. Zhang, Aggregation-induced emission block copolymers based on ring-opening metathesis polymerization, *RSC Advances*, 2014, **4**, 51194-51200.
5. N. Vidović, G. Horvat, D. Riva, T. Rinkovec, N. Cindro, V. Tomišić and G. Speranza, Chloride-Assisted Peptide Macrocyclization, *Org. Lett.*, 2020, **22**, 2129-2134.
6. S. J. Lee, S.-H. Cho, K. L. Mulfort, D. M. Tiede, J. T. Hupp and S. T. Nguyen, Cavity-Tailored, Self-Sorting Supramolecular Catalytic Boxes for Selective Oxidation, *J. Am. Chem. Soc.*, 2008, **130**, 16828-16829.
7. V. Percec, M. R. Imam, M. Peterca, D. A. Wilson, R. Graf, H. W. Spiess, V. S. K. Balagurusamy and P. A. Heiney, Self-Assembly of Dendronized Triphenylenes into Helical Pyramidal Columns and Chiral Spheres, *J. Am. Chem. Soc.*, 2009, **131**, 7662-7677.

8. S. Panja, B. Dietrich and D. J. Adams, Controlling Syneresis of Hydrogels Using Organic Salts, *Angew. Chem. Int. Ed.*, 2022, **61**, e202115021.

9. Somobrata Acharya, Sandip BISWAS, Umesh . et al. Propeller Induced Complete Transformation of the Secondary Structure of a Dipeptide on Water Surface Controlled by Chiral Supramolecular Assembly, 20 December 2023, PREPRINT (Version 1) available at Research Square [https://doi.org/10.21203/rs.3.rs-3771832/v1]
